# Population response of intestinal microbiota to acute *Vibrio alginolyticus* infection in half-smooth tongue sole (*Cynoglossus semilaevis*)

**DOI:** 10.3389/fmicb.2023.1178575

**Published:** 2023-05-15

**Authors:** Yaotong Hao, Yaxian Zhao, Yitong Zhang, Yufeng Liu, Guixing Wang, Zhongwei He, Wei Cao, Tian Han, Xun Zhang, Ziying Zhang, Yufen Wang, Chunguang Gong, Jilun Hou

**Affiliations:** ^1^Ocean College, Hebei Agricultural University, Qinhuangdao, China; ^2^Hebei Key Laboratory of the Bohai Sea Fish Germplasm Resources Conservation and Utilization, Beidaihe Central Experiment Station, Chinese Academy of Fishery Sciences, Qinhuangdao, China

**Keywords:** *Vibrio alginolyticus*, half-smooth tongue sole, secondary infection, intestinal vibrios, metagenomics

## Abstract

**Introduction:**

Vibriosis causes enormous economic losses of marine fish. The present study investigated the intestinal microbial response to acute infection of half-smooth tongue sole with different-dose *Vibrio alginolyticus* within 72 h by metagenomic sequencing.

**Methods:**

The inoculation amount of *V. alginolyticus* for the control, low-dose, moderate-dose, and high-dose groups were 0, 8.5 × 101, 8.5 × 104, and 8.5 × 107 cells/g respectively, the infected fish were farmed in an automatic seawater circulation system under a relatively stable temperature, dissolved oxygen and photoperiod, and 3 ~ 6 intestinal samples per group with high-quality DNA assay were used for metagenomics analysis.

**Results:**

The acute infections with *V. alginolyticus* at high, medium, and low doses caused the change of different-type leukocytes at 24 h, whereas the joint action of monocytes and neutrophils to cope with the pathogen infection only occurred in the high-dose group at 72 h. The metagenomic results suggest that a high-dose *V. alginolyticus* infection can significantly alter the intestinal microbiota, decrease the microbial α-diversity, and increase the bacteria from Vibrio and Shewanella, including various potential pathogens at 24 h. High-abundance species of potential pathogens such as *V. harveyii*, *V. parahaemolyticus*, *V. cholerae*, *V. vulnificus*, and *V. scophthalmi* exhibited significant positive correlations with *V. alginolyticus*. The function analysis revealed that the high-dose inflection group could increase the genes closely related to pathogen infection, involved in cell motility, cell wall/ membrane/envelope biogenesis, material transport and metabolism, and the pathways of quorum sensing, biofilm formation, flagellar assembly, bacterial chemotaxis, virulence factors and antibiotic resistances mainly from Vibrios within 72 h.

**Discussion:**

It indicates that the half-smooth tongue sole is highly likely to be a secondary infection with intestinal potential pathogens, especially species from *Vibrio* and that the disease could become even more complicated because of the accumulation and transfer of antibiotic-resistance genes in intestinal bacteria during the process of *V. alginolyticus* intensified infection.

## 1. Introduction

Intestinal microbiota, an assortment of microorganisms residing in the animal’s gastrointestinal tract, is crucial for the host’s survival because intestinal microbiota regulate metabolism, enhance nutrients absorption, maintain the intestinal epithelial barrier, and adjust the immune system (Kurilshikov et al., 2017). Intestinal microbes can generally protect the host by inhibiting the growth and proliferation of pathogens. However, the intestinal microbiota’s composition and function can be profoundly altered in many disease settings such as pathogen infections. Many intestinal autochthonous microbes belong to opportunistic pathogens. Opportunistic pathogens may occasionally cooperate with primary pathogenic microbes to aggravate the severity of the host disease through coinfection. In marine fishes such as flounder, red sea bream, and yellowtail, *Flexibacter maritimus* is the primary pathogen and secondary infection with vibrios; other opportunistic pathogens are common in severely diseased fish infected with *F. maritimus* (Riichi and Kenji, 1998). In the hybrid red tilapia (*Oreochromis niloticus* × *Oreochromis mossambicus*) chronically infected with *Francisella noatunensis* subsp. *Orientalis*, *Streptococcus agalactiae* infection caused an onset of mortality that was more rapid and occurred at a significantly higher rate than in fish without the secondary infection (Sirimanapong et al., 2018; Abdel-Latif et al., 2020). In striped catfish (*Pangasianodon hypophthalmus*), coinfection of *Flavobacterium columnare* and *Edwardsiella ictaluri* caused significantly high cumulative mortality than a single infection with either *F. columnare* or *E. ictaluri* at the same dose of bacteria (Dong et al., 2015). Although multiple opportunistic pathogens are responsible for disease outbreaks among aquatic animals, the potential risks of coinfection caused by intestinal microbiota under a pathogen infection are still limited cognition, especially in fish.

Half-smooth tongue sole (*Cynoglossus semilaevis*), a kind of large bottom fish with warm water, mainly distributed in the Yellow Sea and Bohai Sea of China (Lin et al., 2021; Wang et al., 2021b). The recent increase in culture density and quantity and environment deterioration has led to the frequent occurrence of vibriosis in half-smooth tongue sole, which causes enormous economic losses (Zhang et al., 2020; Gong et al., 2021; Qi et al., 2021; Zhao et al., 2022). Notably, many pathogenic vibrio species can exist naturally as symbiotic groups in the intestines of healthy fish.

*Vibrio alginolyticus*, as a halophilic, thermophilic, and facultative anaerobic marine *vibrio*, is widely distributed throughout the world’s marine estuaries, coastlines, and aquatic environments, and typically dominates vibrio communities because of its high abundance (Narracci et al., 2014). Moreover, *V. alginolyticus* is a common intestinal bacterial species in marine fish. The pathogenic effects of *V. alginolyticus* infection or proliferation primarily cause damage to host cells and tissues and disrupt normal metabolisms and body functions, either locally or systemically, by its metabolites (Yeh et al., 2009; Fu et al., 2021). *V. alginolyticus* infects marine fish also mainly through abrasions on the fish’s surface, and the low pH of the gastric fluid of seawater carnivorous fish considered to have an inhibitory effect on *V. alginolyticus* (Emam et al., 2019; Huang et al., 2019; Suyanti et al., 2021). Hemorrhagic septicemia, enteritis, and skin ulceration are the typical symptoms of *V. alginolyticus* infection in fish (Wang et al., 2016; Mohamad et al., 2019; Liu et al., 2020). The pathogenesis of *V. alginolyticus* can be attributed to several virulence factors, such as proteases, exotoxins, and siderophores (Liu et al., 2017; Cai et al., 2018; Dong et al., 2020). So far, little is known about the dynamic response and function change of intestinal microbes under *V. alginolyticus* infection in fish.

The present study examined the response mechanism of intestinal microbiota to acute *V. alginolyticus* infection by metagenomic sequencing in the half-smooth tongue soles. The results of this study demonstrated the rapid change of intestinal microbial group and abundance, as well as the possible gene-level coinfection associated with *V. alginolyticus* outbreak.

## 2. Materials and methods

### 2.1. Source and acclimation of half-smooth tongue sole

This experiment was conducted at Beidaihe Central Experiment Station, Chinese Academy of Fishery Sciences, Qinhuangdao, China. About 300 fish (length: 31.47 ± 2.62 cm; bodyweight: 121.36 ± 6.53 g) were obtained from commercial suppliers in Tianjin, China. The fish were temporarily reared for 2 weeks in 3,000 L aquariums with a density of 100 fish per aquarium while being fed a commercial diet with 48% protein content, 10% lipid content and 2% fiber content (Cat. No: 6#, Santong Bio-engineering Co. Ltd., Weifang, China). Fish were fed 2.5% of their body weight at 9 a.m. and 4 p.m. each day for half an hour, and maintained under a stable photoperiod (12 h light, 12 h dark). The aquaculture system was an automatic seawater circulation system with a water exchange rate of about 65 L/h, and the dissolved oxygen concentration was 8.20 ± 0.58 mg/L in all aquariums. The water temperatures of the aquariums ranged from 23 to 25°C.

### 2.2. *Vibrio alginolyticus* challenge test

*Vibrio alginolyticus* was obtained from the diseased half-smooth tongue sole with enteritis and ulcers on the skin surface (Supplementary Figure S1). To ensure accurate infective dose, intraperitoneal injection was used. Through intraperitoneal injection, the median lethal dose (LD50) of *V. alginolyticus* strain within 72 h was found to be 8.5 × 10^4^ cells/g. *V. alginolyticus* strain was subcultured in the BTB medium, and the living bacterial counts were performed using the TCBS agar plate. Both media were obtained from Qingdao Hope Bio-Technology Co. Ltd., China. The bacterial count was calculated quantitatively by integrating the hemocytometer and turbidimetric method at 600 nm and was verified using plate count. *V. alginolyticus* culture broth was centrifuged at 12,000 × *g* for 10 min and washed three times with sterile phosphate-buffered saline (PBS) solution. The bacteria were re-suspended in PBS to adjust the density to 1 × 10^5^, 1 × 10^8^, and 1 × 10^11^ cells/mL.

To simulate different stages of proliferation of *V. alginolyticus*, three treatment groups with different pathogen loads were studied. The inoculation amounts of the control (C), low-dose (L), moderate-dose (M), and high-dose (H) groups were 0, 8.5 × 10^1^, 8.5 × 10^4^, and 8.5 × 10^7^ cells/g, respectively. The concentrated bacterial suspension was intraperitoneally administered to the fish through injection. One hundred and sixty fish (40 fish per group) were randomly selected for the pathogen challenge test. For blood and intestinal tissue sampling, 40 fish from the C, L, M, and H groups were placed in four separate aquariums measuring 1.2 m × 1 m × 1 m with an actual water volume of 720 L.

### 2.3. Sampling

After intraperitoneal injection, 6 fish from each group were randomly picked out and anesthetized with 100 mg/L eugenol at 24 and 72 h. Before sampling, the fish’s body was sterilized with 70% alcohol. Blood was aseptically collected from the caudal vein of fish with a disposable sterile syringe and immediately transferred into collection tubes containing heparin anticoagulant for hematology analysis. The intestinal contents, located in the sections from the foregut to the anus, were collected with a medical-grade swab, rapidly frozen in liquid nitrogen, and stored at −80°C before microbial genomic DNA extraction.

### 2.4. Hematology analysis

The leukocytes (lymphocyte, monocyte, neutrophil, eosinophil, and basophil) of each sample were determined using a UniCel DxH 800 Coulter hematology analyzer (Beckman Coulter, CA, United States) following the manufacturer’s protocols.

### 2.5. Metagenomic sequencing

Total genomic DNA was extracted from the intestinal contents (approximately 200 mg for each fish) using the QIAamp DNA extraction kit for stool (QIAGEN, Hilden, Germany) following the manufacturer’s instructions. The level of DNA degradation degree and potential contamination was monitored on 1% agarose gels. DNA concentration was measured using the Qubit® dsDNA Assay Kit in Qubit® 2.0 fluorometer (Life Technologies, CA, United States). The input material for the DNA sample preparations consisted of 1 μg DNA per sample. Sequencing libraries were generated using the NEBNext® Ultra™ DNA Library Prep Kit for Illumina (NEB, United States) following the manufacturer’s recommendations. The final 41 sample libraries of 8 groups (3 ~ 6 per group) reached grade A for metagenomic sequencing analysis. On an Illumina HiSeq platform, the library preparations were sequenced and paired-end reads were generated.

### 2.6. Data processing

#### 2.6.1. Preprocessing of data

The Raw Data obtained from the Illumina HiSeq sequencing platform using Readfq (V8^1^) was processed to acquire the Clean Data for subsequent analysis. The specific steps were as follows: (a) the reads containing low quality bases (default quality threshold value ≤38) above a certain portion (default length of 40 bp) were removed; (b) the reads in which the N base had reached a certain percentage (default length of 10 bp) were removed; (c) the reads which shared the overlap above a certain portion with Adapter (default length of 15 bp) were removed. The Clean Data was to blast with the host genome (Cse_v1.0 from NCBI database) using Bowtie2.2.4 software (Bowtie2.2.4^2^) to filter host-originated reads. The Clean Data of each sample were assembled using SOAPdenovo software (V2.04^3^) with the parameters of -d 1, −M 3, −R, −u, -F, -K 55 (Scher et al., 2013; Qin et al., 2014; Brum et al., 2015; Feng et al., 2015). All unutilized reads from the forward step of all samples were combined, and the SOAPdenovo (V2.04) software was used for mixed assembly with the same parameters as a single sample assembly. The mixed assembly Scaffolds were to break from the N connection to obtain Scaftigs. For statistical analysis, the fragments of Scaftigs shorter than 500 bp, whether generated from the single or mixed assembly, were filtered out.

#### 2.6.2. Gene prediction and abundance analysis

The Scaftigs (≥500 bp) were predicted for the ORF through the MetaGeneMark (V2.10^4^) software. Based on the predicted ORF, the CD-HIT software (V4.5.8^5^) was adopted to eliminate redundancy and obtain the unique initial gene catalog. The Clean Data of each sample was mapped to the initial gene catalog using Bowtie2.2.4. The number of reads, which genes successfully mapped in each sample, was obtained with a set of parameters (Li et al., 2014; Qin et al., 2014). Each sample’s reads with a count of ≤2 were filtered out, and a gene catalog (Unigenes) was obtained for subsequent analysis. Finally, an average of 4,362 scaftigs were assembled per sample with an average length of 1,263 bp. The length of N50 and N90 was 4,931 bp and 549 bp, respectively. A total of 226,536 ORFs were predicted by MetaGeneMark from Scaftigs (≥500 bp), with an average of 5,525 ORFs per sample. A total of 110,690 non-redundant genes were eventually obtained by eliminated redundancy of ORFs. These genes were with a total length of 65.94 mbp, an average length of 595.69 bp, and a GC percentage of 44.32%. The numbers of non-redundant genes of control, L and M groups at 24 and 72 h were significantly lower than that in H group at 72 h (*p* < 0.05). The number of genes in H group at 24 h with large intra-group differences was no significantly difference with other groups (Supplementary Figure S2).

#### 2.6.3. Gene annotation

For taxonomy annotation, the DIAMOND software (V0.9.9^6^) was used to blast the Unigenes to the sequences of bacteria, fungi, archaea, and viruses that were extracted from the NR database (V2018-01-02^7^) of NCBI. The LCA algorithm was applied to the systematic classification of the MEGAN software to ensure the species annotation information of sequences. The DIAMOND software (V0.9.9) was then adopted to blast the Unigenes to a functional database with the parameter setting of blastp, −e 1e-5 (Li et al., 2014; Feng et al., 2015). The functional database excluded the KEGG database (V2018-01-01^8^), the eggNOG database (V4.5^9^), and the CAZy database (V201801^10^). The Resistance Gene Identifier (RGI) software was used to align the Unigenes to the CARD database^11^ with the parameter setting of blastp, e value ≤1e-30 (Jia et al., 2017). The results of functional and resistance gene annotations were summarized with the taxonomy annotation results to clarify the role and species information of the Unigenes. Gene annotation results showed that 63.22% of Unigenes had been taxonomy annotation, 55.97% finished the KEGG functional annotation, and 51.73% finished eggNOG functional annotation.

### 2.7. Quantitative analysis of *Vibrio* genus and *groEL* gene in intestine

To quantify the levels of *Vibrio* genus in intestine, the transcriptional levels of 16S rRNA were quantitatively analyzed by qPCR with *Vibrio*-specific primers (V27F: 5′-AGA GTT TGA TCC/ATG GCT CAG-3′; V744R: 5′-CAT CTG AGT GTC AGT G/AT CTG−3′) (Liu et al., 2006). When bacterial cells invade host tissue, the *groEL* gene is induced to express at a markedly higher level to protect bacterial cells from the host environment. To estimate the invasion of pathogen in the tissue of intestine, the transcriptional levels of *groEL* gene were quantitatively analyzed by qPCR with *V. alginolyticus*-specific primers (F-groEL: 5′-GAT TCG GTG AAG AGA TGA TCT C-3′; R-groEL: 5′- TCT TCG TTG TCA CCC GTT AGG TGA-3′) (Ahmed et al., 2016). Total RNA was extracted from by Trizol regent. First-strand cDNAs were obtained using a random hexamer primer and the ReverTr Ace kit (Toyobo, Japan). qPCR was carried out using Quantagene q255 qPCR system (KUBO Technology, China). Each assay was performed in triplicate in a reaction mixture containing 5 μl of TB Green Premix Ex Taq II (Tli RNaseH Plus), each 0.5 μl of forward and reverse primer, 0.5 μl of cDNA and 3.5 μl of RNase-free dH2O. β-actin gene was used as a reference gene (F: 5′-GCT GTG CTG TCC CTG TA-3′; R: 5′-GAG TAG CCA CGC TCT GTC-3′). The PCR cycling condition was: 94°C for 2 min, denaturation at 94°C for 30 s, extension at 55°C for 20 s, for a total of 40 cycles. The accuracy and specificity of the PCR products were determined by a dissolution curve, and the relative expression level of mRNA was calculated using the 2^-ΔΔCt^ method (Livak and Schmittgen, 2001).

### 2.8. Statistical analysis

The basic information statistic, core-pan gene analysis, correlation analysis of samples, and Venn figure analysis of the number of genes were all based on the abundance of each gene in each sample in the gene catalog. The exhibition of abundance cluster heat map and non-metric multidimensional scaling (NMDS) decrease-dimension analysis were based on the abundance table of each taxonomic hierarchy by R. Principal co-ordinates analysis (PCoA) based on Bray–Curtis distances was applied to visualize the differences between microbial structures; Analysis of molecular variance (AMOVA) based on weighted-unifrac distances was used in R. Linear discriminant analysis effect Size (LEfSe) analysis was conducted with the LEfSe software (the default LDA score was 3). Permutational multivariate analyses of variance (PERMANOVA) were performed to test the significance of differences among microbial structures. The mean comparison of data among three or more groups was analyzed using a one-way analysis of variance (ANOVA) followed by Tukey’s test for multiple comparisons with SPSS (V18.0). For statistically significant differences, *p* < 0.05 was required. All data are expressed as mean ± standard deviation.

## 3. Results

### 3.1. The effect of *Vibrio alginolyticus* infection on the hematology index

At 24 h, leukocyte count was no significantly differences between infected groups, but the proportion of different-leukocyte types did (Table 1). Compared to the C group, the atypical lymphocyte and monocyte proportions were higher and the lymphocyte proportion was lower in the H group (*p* < 0.05), the atypical lymphocyte proportion was higher in the M group (*p* < 0.05), and the monocyte proportion was higher in the L group (*p* < 0.05). At 72 h, the leukocyte count, lymphocyte proportion, and atypical lymphocyte proportion were significantly lower and the neutrophil and monocyte proportions were significantly higher in the H group than those in the C group (*p* < 0.05). Moreover, the proportions of eosinophils and basophils increased in the H group, but no significant difference was observed compared with the C group (*p* > 0.05).

**Table 1:** Leukocyte statistical analysis.

Time	Index	Groups				*p-*values
		C	L	M	H	
24 h	Leukocyte (10^4^/mL)	1.06 ± 0.03	1.04 ± 0.09	1.15 ± 0.02	1.10 ± 0.03	0.461
	Lymphocyte (%)	97.13 ± 0.47^a^	95.37 ± 0.49^a^	79.8 ± 11.62^ab^	61.47 ± 5.02^b^	0.011
	Atypical lymphocytes (%)	7.10 ± 0.91^a^	6.25 ± 0.97^a^	13.29 ± 2.06^b^	16.15 ± 1.01^b^	0.002
	Monocyte (%)	2.73 ± 0.48^a^	4.47 ± 0.46^b^	18.27 ± 10.04^ab^	32.3 ± 2.13^b^	0.010
	Neutrophil (%)	0.13 ± 0.03	0.17 ± 0.03	1.90 ± 1.56	6.13 ± 2.81	0.090
	Eosinophil (%)	0.00 ± 0.00	0.00 ± 0.00	0.03 ± 0.03	0.10 ± 0.10	0.528
	Basophil (%)	0.00 ± 0.00	0.00 ± 0.00	0.00 ± 0.00	0.00 ± 0.00	NA
72 h	Leukocyte (10^4^/mL)	0.98 ± 0.11^a^	1.01 ± 0.03^a^	0.94 ± 0.02^a^	0.56 ± 0.16^b^	0.042
	Lymphocyte (%)	94.57 ± 1.68^a^	92.87 ± 1.11^a^	98.30 ± 0.25^a^	17.20 ± 7.29^b^	0.000
	Atypical lymphocytes (%)	9.50 ± 1.60^a^	8.33 ± 0.64^a^	4.97 ± 0.18^ab^	3.12 ± 1.07^b^	0.008
	Monocyte (%)	5.13 ± 1.60^ab^	4.53 ± 0.58^ab^	1.40 ± 0.10^a^	18.60 ± 6.20^b^	0.023
	Neutrophil (%)	0.30 ± 0.12^a^	2.60 ± 1.14^a^	0.30 ± 0.15^a^	37.53 ± 5.53^b^	0.000
	Eosinophil (%)	0.00 ± 0.00	0.00 ± 0.00	0.00 ± 0.00	26.6.0 ± 13.73	0.060
	Basophil (%)	0.00 ± 0.00	0.00 ± 0.00	0.00 ± 0.00	0.50 ± 0.40	0.272

Values represent means and standard errors of five replicates (means ± SE; *n* = 5). Values in the same row that had different superscripts are significantly different at *p* < 0.05 based on Tukey’s test. *p-*value showed the result of one-way analysis of variance (ANOVA) among treatments. C, the control group; L, the inoculation amount of the pathogen was 8.5 × 10^1^ cells/g; M, the inoculation amount was 8.5 × 10^4^ cell/g; H, the inoculation amount was 8.5 × 10^7^ cell/g.

### 3.2. The 16S rRNA and *groEL* gene expression in intestine

The level of 16S rRNA from *Vibrio* genus in intestine was showed that at 24 and 72 h, significantly higher levels of 16S rRNA were only found in group H than that in group C (*p* < 0.05) (Supplementary Figure S3A). The effects of challenge test on the loads of *V. alginolyticus* in intestine were quantified by the transcriptional levels of *groEL* gene with *V. alginolyticus*-specific primers. At 24 and 72 h, *groEL* gene expression with a high level was detected only in the high-dose group (Supplementary Figure S3B).

### 3.3. The response of intestinal microbiota to pathogen infection

**Figure 1:**
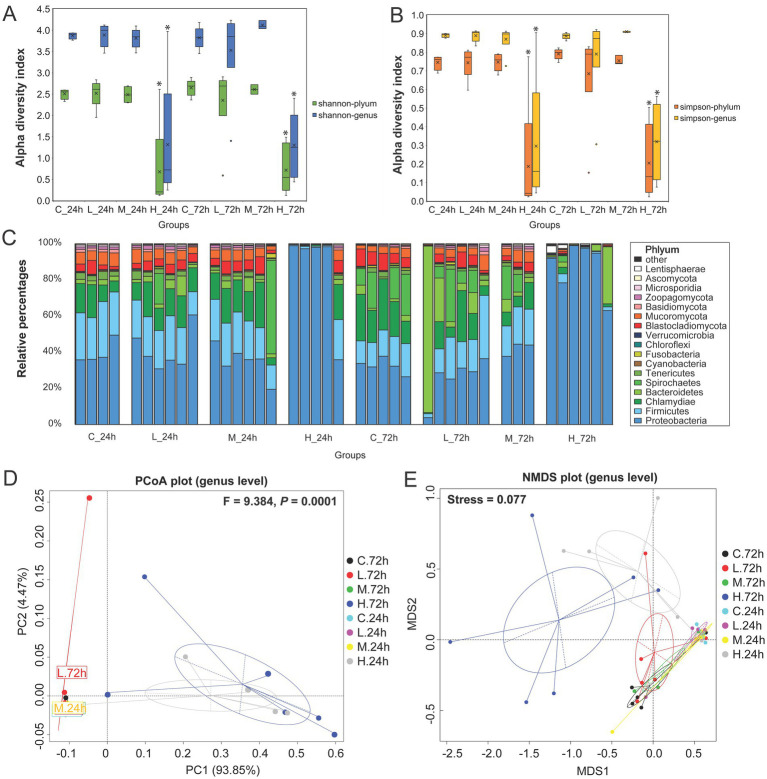
Microbial community composition analysis in the intestine of half-smooth tongue sole infected with varying doses of *Vibrio alginolyticus*. For the groups, C represents the control group (0 cells/g), L represents the low-dose challenge test group (8.5 × 10^1^ cells/g), M represents the medium-dose challenge test group (8.5 × 10^4^ cells/g), and H represents the high-dose challenge test group (8.5 × 10^7^ cells/g) (*n* = 3 ~ 6 per group). **(A)** Shannon index at the phylum and genus levels exhibits the intestinal microbial alpha diversity index. “*” Indicates a significant difference with other groups (*p* < 0.05). **(B)** Simpson indexes at the phylum and genus levels exhibit the intestinal microbial alpha diversity index. “*” indicates a significant difference with other groups (*p* < 0.05). **(C)** Relative abundance of gut microbial taxa at the phylum level. **(D)** PCoA analysis based on Bray–Curtis distances was used to demonstrate the effects of pathogens on the microbial community structure in the genus after 72 h. (*p* < 0.05 indicates that there are significant differences in community structure between groups). **(E)** NMDS analysis was used to exhibit the degree of difference based on the distance between sample points at the genus level. (Stress <0.2 indicates that the NMDS analysis is reliable).

Based on taxonomy annotation results, the microbial α diversity indexes (Shannon and Simpson) of the H group at the phylum and genus levels were significantly lower than those of the C, L, and M groups at 24 and 72 h, but no significant difference was observed in the H group at 24 and 72 h (Figures 1A,B). Besides bacteria, eukaryotes constitute a portion of the predominant microbial composition. For bacteria, Proteobacteria was the dominant phylum in all groups, followed by Firmicutes, Chlamydiae, Bacteroidetes, and Spirochaetes. For eukaryotes, the dominant phyla were Blastocladiomycota and Mucoromycota, of which the proportions were relatively low in the H group at 24 and 72 h (Figure 1C). The LEfSe analysis demonstrated that Flavobacteriaceae was increased in the L group at 72 h, Glomeraceae increased in the M group at 72 h, and Shewanellaceae and Vibrionaceae increased in the H group at 72 h (Supplementary Figure S4A). Moreover, *Vibrio* levels were relatively high in the H group at both 24 and 72 h, with distinct species at each time point (Supplementary Figure S4B). At genus level, PCoA and NMDS demonstrated that *V. alginolyticus* infection had a significant effect on the microbial community structure (PERMANOVA *F* = 9.384, *p* = 0.0001), and the H group was significantly separated from other groups at 24 and 72 h (Figures 1D,E).

**Figure 2:**
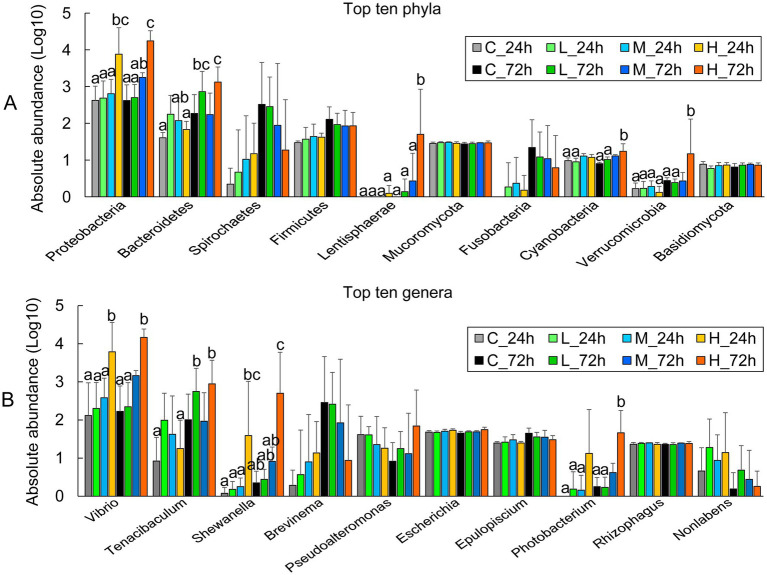
The abundance of major microbial groups in the intestine of half-smooth tongue sole after different-dose *Vibrio alginolyticus* infection at 24 and 72 h. **(A)** The absolute abundance of the top 10 phyla is expressed as a Log10 of the number of genes in the phylum-level annotation. **(B)** The absolute abundance of the top 10 genera is expressed as a Log10 number of genes in a genus-level annotation. The error bars represent the standard error of the mean. The significant differences (*P* < 0.05) among the treatments and controls are indicated by the different lowercase letters above the bars.

Comparing the abundances of the top 10 phyla revealed that the H group contained higher proteobacteria at 24 and 72 h than the C, L, and M groups. Bacteroidetes, Lentisphaerae, Cyanobacteria, and Verrucomicrobia were significantly more abundant in the H group at 72 h (Figure 2A). The abundance of *Vibrio* in the H group was significantly higher than that in the C and L groups at 24 and 72 h, and higher than that in the M group at 24 h, but no significant difference with the M group at 72 h. The abundance of *Tenacibaculum* was significantly higher in the H group at 72 h than that at 24 h. The abundance of *Shewanella* in the H group was significantly higher than that in the C, L, and M groups at 24 and 72 h (Figure 2B).

### 3.4. The relationship among intestinal microbiomes

**Figure 3:**
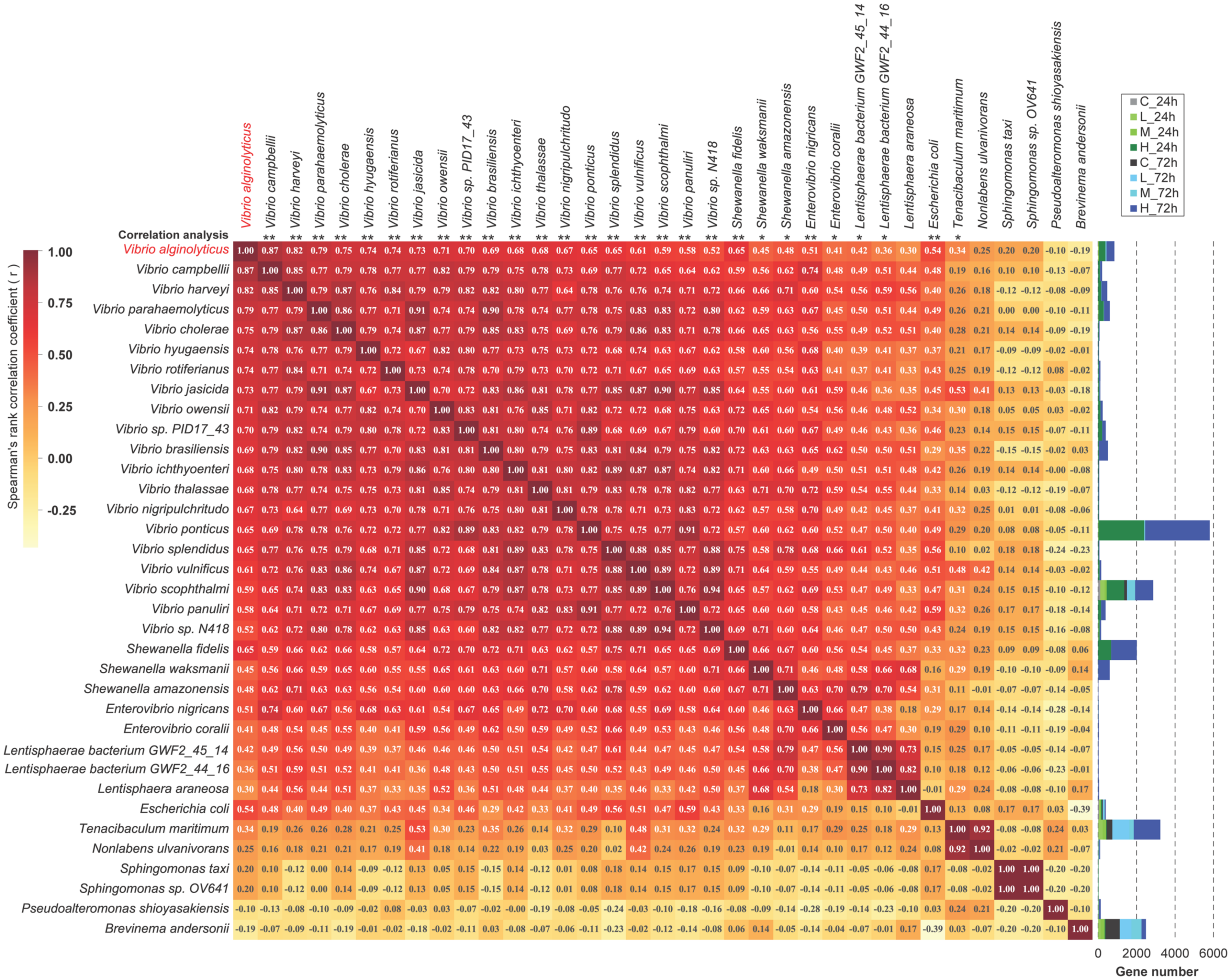
The correlation analysis among the top 35 species. Spearman’s correlation was used to analyze the correlation between any two species. The value of Spearman’s correlation *r* is indicated in the corresponding square. A positive value indicates positive correlation, while a negative value indicates negative correlation. The significance of the similarity between each species and *Vibrio alginolyticus* is marked at the top of the picture, and “*” means “*p* < 0.05,” and “**” means “*p* < 0.001.”

The correlation analysis of the top 35 bacterial species revealed that bacterial species from the same genus were positively associated with each other, including 20 species from *Vibrio* (Spearman, *r* ≥ 0.52), 3 of *Shewanella* (*r* ≥ 0.66), 2 of *Enterovibrio* (*r* ≥ 0.66), 3 of *Lentisphaerae* (*r* ≥ 0.73) and 2 of *Sphingomonas* (*r* = 1) (Figure 3). The significance analysis demonstrated that *V. alginolyticus* had extremely significant positive correlations with other vibrios, including *V. Harveyii*, *V. parahaemolyticus*, *V. cholerae*, *V. vulnificus*, *V. scophthalmi*, etc. (*p* < 0.001). Moreover, *V. alginolyticus* had extremely significant positive correlations with *Shewanella fidelis*, *Enterovibrio nigricans*, and *Escherichia coli* (*p* < 0.001) and significant positive correlations with *S. waksmanii*, *S. amazonensis*, *Enterovibrio coralii*, *Tenacibaculum maritimum*, *Lentisphaerae bacterium GWF2_45_14* and *GWF2_44_16* (0.001 < *p* < 0.05). *V. alginolyticus* had negative correlations with *Pseudoalteromonas shioyasakiensis* (*r* = −0.10) and *Brevinema andersonii* (*r* = −0.19) but was not significant (Figure 3).

### 3.5. The shift of intestinal microbial function with pathogen infection

**Figure 4:**
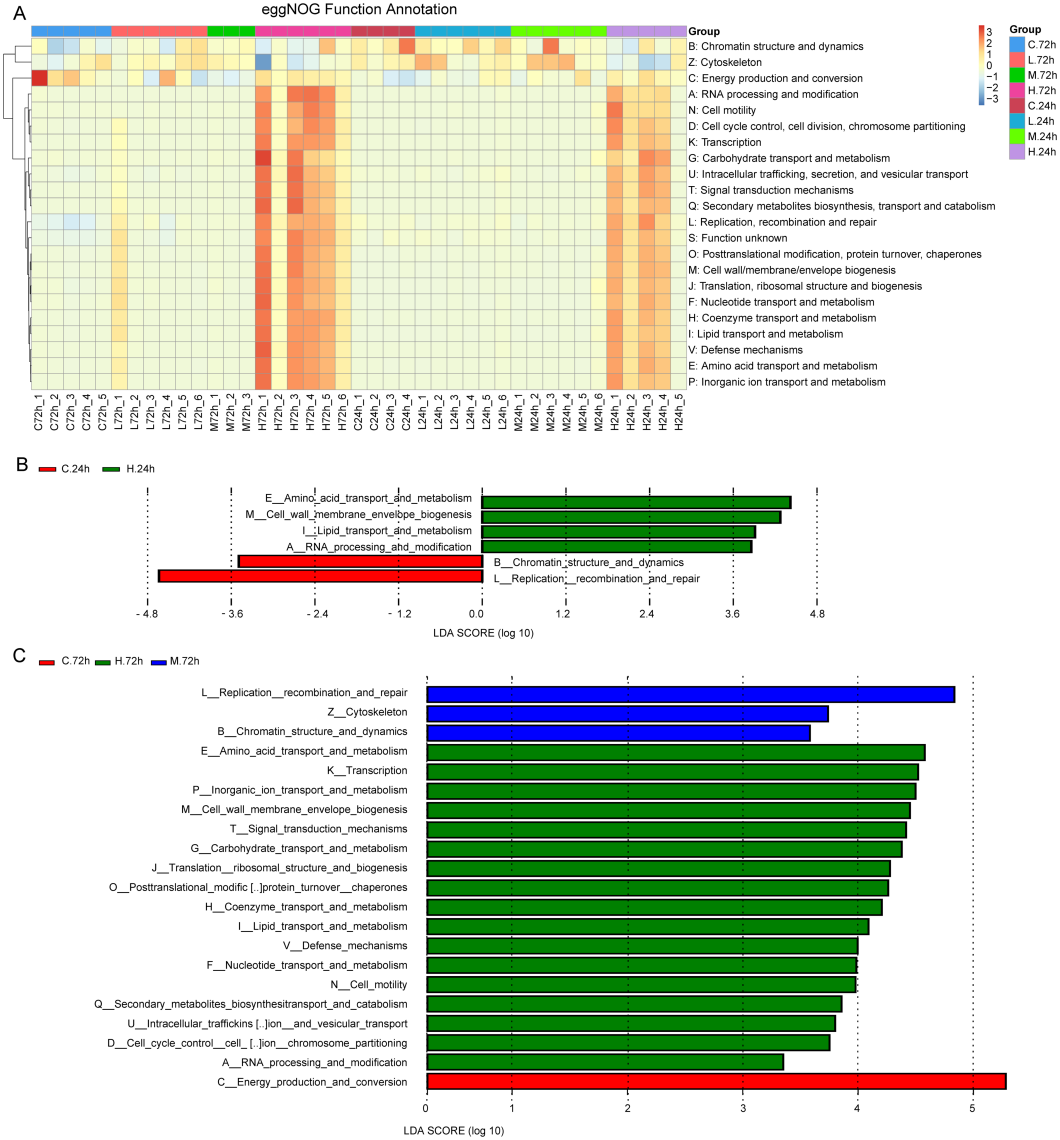
The function analysis based on eggNOG function annotation. **(A)** Heat map shows eggNOG function annotations. The cluster tree on the left is the functional cluster tree. The value corresponding to the heat map color is the result of the standardized relative abundances of each row. **(B)** Histogram of the LDA value distribution of function genes with differences at 24 h after *Vibrio alginolyticus* infection. **(C)** Histogram of LDA value distribution of function genes with differences at 72 h after *V. alginolyticus* infection. The annotated function whose LDA score was greater than the set value (the default value was 3) was the biomarker with the statistical difference between groups.

Supplementary Table S1 presents the numbers of Unigenes with functional annotations. The results of eggNOG function annotation revealed that the numbers of genes involved in cell motility, cell wall/membrane/envelope biogenesis, and material transport and metabolism (carbohydrate, lipid, amino acid, nucleotide, coenzyme, inorganic ion) increased in samples from the H group at 24 and 72 h after challenge test, compared with those from the C, L, and M groups (Figure 4A). The LEfSe analysis showed that the genes involved in lipid and amino acid transport and metabolism, cell wall/membrane/envelope biogenesis, and RNA processing and modification significantly increased in the H group at 24 h (Figure 4B). Besides the increased genes at 24 h, the genes involved in the metabolism of carbohydrate, nucleotide, coenzyme, inorganic ion, and secondary metabolites also significantly increased in the H group at 72 h. The genes involved in replication, recombination, and repair significantly increased in the M group at 72 h. Among the treatment groups, the most genes involved in energy production and conversion were found in the C group at 72 h (Figure 4C).

**Figure 5:**
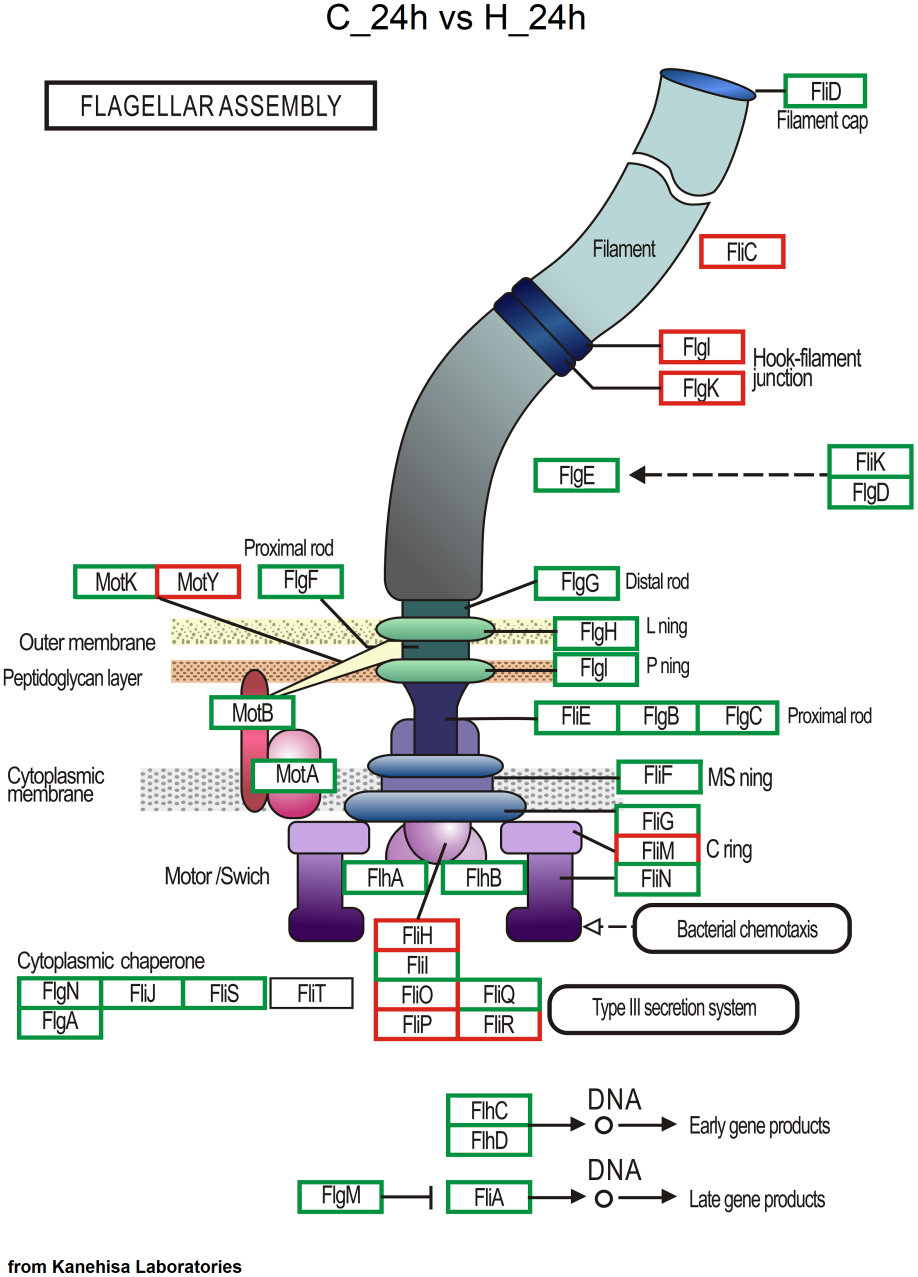
Genes involved in flagellar assembly based on KEGG pathway annotation. The red box represents enzymatic reactions common to both groups, the blue box represents enzymatic reactions unique to the C group at 24 h, and the green box represents enzymatic reactions unique to the H group at 24 h.

As determined from KEGG pathway annotation, the numbers of genes involved in microbial community response, including the pathways of ABC transporter, two−component system, quorum sensing, biofilm formation-*Vibrio cholerae*, bacterial chemotaxis, and flagellar assembly (Supplementary Figure S5) and σ factors (Supplementary Table S2), increased in the H group at 24 and 72 h after challenge test. Most of these function genes were from *Vibrio*, followed by *Shewanella* and *Pseudoalteromonas* (Supplementary Table S3). The maps of the two−component system, ABC transporter, quorum sensing, biofilm formation-*Vibrio cholerae*, and bacterial chemotaxis of the C vs. H groups at 24 h are shown in Supplementary Figures S6–S10. For flagellar assembly, 709 annotated Unigenes included 426 genes from *Vibrio*, 153 genes from *Shewanella*, 43 genes from *Pseudoalteromonas*, and 25 genes from *Brevinema*. The number of Unigenes for flagellar assembly increased as the pathogen dose increased. Compared with the C group, more Unigenes for flagellum assembly were annotated in the H group at 24 h (Figure 5), and no difference was observed between H group samples at 24 and 72 h (Supplementary Figure S11). Moreover, the potential virulence genes of *Vibrio* such as *flaA* (flagella basal body P-ring formation protein FlgA, 20 Unigenes), *fliC* (flagellin, 78), *fur* (ferric uptake regulator, 11), *ompW* (outer membrane protein OmpW, 15), *ompU* (outer membrane protein OmpU, 23), *colA* (collagenase, 26), *toxR* (cholera toxin transcriptional activator, 10), *toxS* (transmembrane regulatory protein ToxS, 7), *hppD* (4-hydroxyphenylpyruvate dioxygenase, 14) and *hap* (vibriolysin, 13) were annotated, with most belonging to the H group (Supplementary Table S4).

The results of CAZy annotation revealed an increase in the number of Unigenes for polysaccharide-related enzymes in the H group at 24 and 72 h, such as lipopolysaccharide N-acetylglucosaminyltransferase (EC 2.4.1.56), hyaluronan synthase (EC 2.4.1.212), chitin synthase (EC 2.4.1.16), and chitin oligosaccharide synthase (EC 2.4.1.-) (Supplementary Figure S12).

### 3.6. The change of antibiotic-resistance genes with pathogen infection

**Figure 6:**
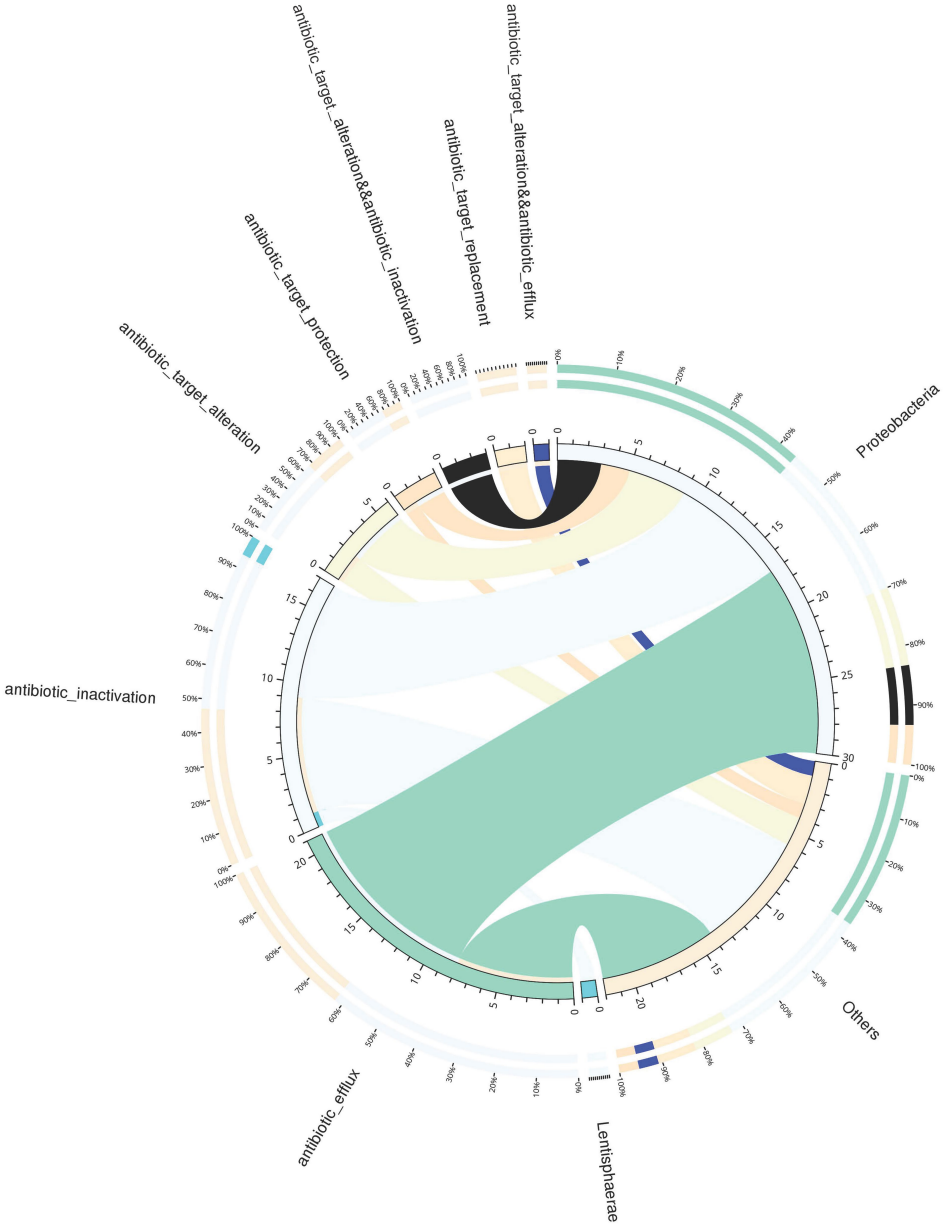
Overview circle diagram of resistance mechanisms and species. The circle diagram was divided into two parts, with phylum-level species information on the right and resistance mechanism information on the left. For the inner circle, the left is the sum of the number of resistance genes containing the resistance mechanism in the species, and the right is the sum of the number of resistance genes contained in the species with different resistance mechanisms. For the outer circle, the left side shows the relative proportion of resistance genes in each species to its resistance mechanism, and the right side shows the relative proportion of resistance genes in each resistance mechanism to the resistance genes from the species it belongs to.

**Figure 7:**
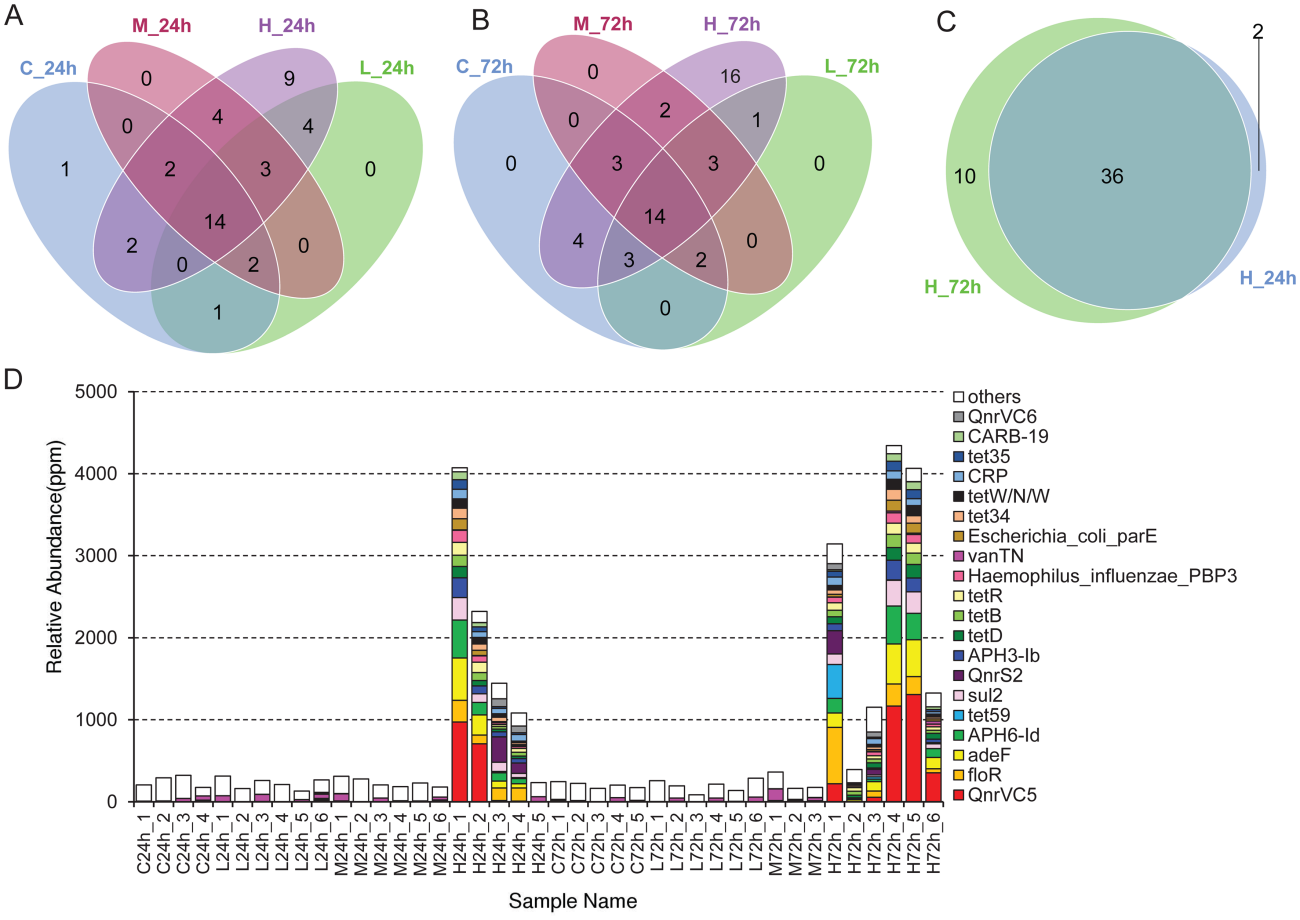
The analysis of antibiotic-resistance genes. **(A)** Venn diagram analysis of antibiotic-resistance genes in different treatments at 24 h. **(B)** Venn diagram analysis of antibiotic-resistance genes in different treatments at 72 h. **(C)** Venn diagram analysis of antibiotic-resistance genes in high-dose infection group between 24 and 72 h. **(D)** Histogram of the abundance of different AROs in each sample. The unit in ppm is the result of scaling up the original relative abundance data by a factor of 10^6^.

A total of 66 antibiotic-resistance genes were annotated based on the CARD database. Cluster analysis showed that these genes were divided into 49 ARO groups (Supplementary Table S5). The overview circle diagram showed that intestinal microbial antibiotics resistance genes were mainly derived from the class of Gammaproteobacteria, and the main AROs were QnrVC5, adeF, and floR (Figure 6). The Venn diagram revealed that the number of antibiotic-resistance genes in the H group was much higher than those in the C, L, and M groups at 24 and 72 h after pathogen infection (Figures 7A,B). In the H group, the number of antibiotic-resistance genes at 72 h was higher than that at 24 h (Figure 7C). Furthermore, the relative abundance of antibiotic-resistance genes in the H group increased at 24 and 72 h after pathogen infection (Figure 7D).

## 4. Discussion

*Vibrio* can enter the host’s bloodstream, thereby accelerating pathogen transmission (Gong et al., 2021; Zhang and Li, 2021; Zhang et al., 2022). Bloodborne pathogens that reach the intestine interact with intestinal flora. The intestinal microbiota can resist the invasion of foreign microorganisms through the antagonism between microorganisms for maintaining the stability of the flora structure, which is crucial for the host’s health (Deng et al., 2020; Li et al., 2021; Chen et al., 2022). In this study, the acute infections with *V. alginolyticus* at high, medium, and low doses changed leukocyte proportion in the blood. However, infections with *V. alginolyticus* at medium and low doses had no significant effect on the microbial structure within 72 h. In contrast, high-dose infection significantly altered the intestinal microbiome within 24 h. The quantitative results showed that the *groEL* gene expression happened only in the high-dose infection group within 24 h. The *groEL* gene encodes the GroEL chaperone and plays a vital role in the control of cellular stress in bacterial cells as a housekeeping gene. When bacterial cells invade host tissue, the *groEL* gene is induced to express at a markedly higher level to protect bacterial cells from the host environment. It indicates that the host may has an effective defense against the spread of relatively low-dose pathogens, but this ability may diminish with the increase in the pathogen dose.

Once the structure of the intestinal microbiota has been disrupted with the invasion of pathogenic bacteria, various potential pathogenic bacteria have the opportunity to grow, and co-infection or secondary infection may occur immediately (Kotob et al., 2016; Abdel-Latif and Khafaga, 2020). So, the early stage of infection is the key stage for disease control. In the present study, a high dose of *V. alginolyticus* disrupted the microbial community structure, and many bacterial species belonging to the genera *Vibrio* and *Shewanella* were significantly increased. The correlation analysis revealed that *V. alginolyticus* was significantly positively correlated with 19 other bacteria from *Vibrio* and 3 from *Shewanella*. The increased bacteria of *Shewanella* are typical marine microbial groups, occupying a similar ecological niche with *V. alginolyticus*, and the other *Vibrio* species, in addition to sharing the same habitat, also have more close relatives and metabolic types in common with *V. alginolyticus* (Rutschlin et al., 2017; Binnenkade et al., 2018; Tan et al., 2022). Therefore, once the structure of intestinal microbiota has been broken down, the growth of other normal microbiota, including probiotics, may be inhibited by the expansion and exotoxin secretion of pathogenic bacteria, whereas bacterial groups that have adapted to each other and coexisted with pathogenic bacteria for a long time can explode. Notably, these fast-growing bacterial species were common and potent pathogenic bacteria in flounder, such as *V. Harveyii*, *V. parahaemolyticus*, *V. ichthyoenteri*, *V. vulnificus*, and *V. scophthalmi* (Tang et al., 2019; Zhang et al., 2020; Gong et al., 2021; Qi et al., 2021; Zhao et al., 2022). Therefore, as the process of high-dose *V. alginolyticus* infection or pathogen infection intensifies, the half-smooth tongue sole is highly likely to be co-infected with other pathogens, especially other *Vibrio*.

In addition to the pathogen-induced explosive growth of opportunistic bacteria in the host gut, functional information analysis further revealed the possibility of coinfection. Infection by invading pathogens can also induce otherwise commensal bacteria to become pathogenic (Stevens et al., 2021). Quorum sensing—bacterial cell-to-cell communication with small signal molecules—is known to coordinate various biological activities, including motility, biofilm formation, and virulence factor secretion, to control the virulence of many bacteria, such as *Vibrio* species (Kareb and Aider, 2020; Wang et al., 2021a). A quorum sensing system is therefore necessary for the transition of commensal bacteria to pathogens. Vibrios typically contain multichannel quorum sensing systems, which have been documented to be required for the full virulence of vibrios toward various host organisms (Ball et al., 2017; Lu et al., 2018; Defoirdt, 2019). In the present study, more genes involved in quorum sensing, biofilm formation-*Vibrio cholerae*, and flagellar assembly, mainly from the bacteria of *Vibrio*, were detected in the high-dose pathogen infection group at 24 and 72 h. Therefore, a transition of commensal-to-pathogenic bacteria is becoming active in the gut. Moreover, more genes involved in bacterial chemotaxis from *Vibrio* were also found in the high-dose pathogen infection group. Chemotaxis can control the direction of flagellar rotation and promote the rapid expansion of bacterial populations into previously unoccupied territories (Colin et al., 2021; Takekawa et al., 2021). Chemotaxis can be important for vibrios to locate a favorable environment and colonize a host successfully. Chemotactic *V. alginolyticus* with a single polar flagellum swims smoothly by rotating the flagellar motor counterclockwise in response to attractants; non-chemotactic mutants of *V. anguillarum* are attenuated for infection; non-chemotactic *V. fischeri* are impaired for colonization of the Hawaiian bobtail squid (*Euprymna scolopes*); and *V. coralliilyticus* chemotaxes toward coral mucus (Ushijima and Hase, 2018; Echazarreta and Klose, 2019; Takekawa et al., 2021). Therefore, the potential transition of commensal bacteria into pathogens and enhanced bacterial chemotaxis point to the occurrence of co-infection.

Meanwhile, considerable numbers of virulence genes from *Vibrio*, including *flaA*, *fliC*, *fur*, *ompW*, *ompU*, *colA*, *toxR*, *toxS*, *hppD*, and *hap*, were detected in fish intestines of the high-dose infection group, and antibiotic-resistance genes increased alongside virulence genes. Increased virulence and the advent of antibiotic resistance frequently occur almost simultaneously (Schroeder et al., 2017). The close relationship between increased antibiotic resistance and virulence is intimately tied to the ability of bacteria to communicate through quorum sensing and two-component systems both directly and indirectly (Worthington et al., 2013; Guillard et al., 2016; Haque et al., 2021). Moreover, virulence and antibiotic-resistance genes undergo horizontal gene transfer, which can be facilitated by biofilm formation (Meroni et al., 2019; Arunkumar et al., 2020). Combating the spread of antibiotic resistance is one of the most important problems that plague our society today. To control the spread of antibiotic resistance, the spread of virulence, which is often associated with disease, must be controlled in addition to controlling the use of antibiotics (Schroeder et al., 2017). In the present study, more genes involved in biofilm formation, quorum sensing, and two-component systems were found in the high-dose infection group. We speculate that the numbers of virulence and antibiotic-resistance genes can increase rapidly along with an outbreak of potentially pathogenic bacteria, including other vibrios, caused by the invasion of high-dose *V. alginolyticus*; the horizontal transmission of these virulence and antibiotic-resistance genes between bacteria may be enhanced with more active biofilm formation, quorum sensing, and two-component systems. Increased antibiotic resistance may naturally evolve in response to increased virulence, which undoubtedly poses a great challenge to the treatment of diseases.

In conclusion, the acute infection of high-level *V. alginolyticus* in half-smooth tongue sole could disturb the original intestinal microbiota and lead to the explosive growth of potential intestinal pathogens. Furthermore, antibiotic-resistance genes and virulence genes were found to be increased in intestinal microbiota, which provide new insights for bacterial disease control and disease course prediction in farmed fish. Finally, although different doses have been used to show the differences in the intensity of *V. alginolyticus* infection, this study is still a short-term acute infection experiment. Given the widespread existence of vibrios in the habitat and the long lifespan of half-smooth tongue sole, more studies are needed to further reveal the adaptation and struggle between intestinal microbes and pathogens.

## Data availability statement

The metagenomics raw data have been deposited in the NCBI online repository under the accession number PRJNA938989, and the accession number “PRJNA938989” needs to be provided in the edition of the paper to be published.

## Ethics statement

The animal study was reviewed and approved by the Ethics Committee for Experimental Animals of Hebei Agricultural University, China.

## Author contributions

YH contributed to writing—original draft, investigation, and data analysis of the study. YaZ, YiZ, YL, GW, ZH, WC, TH, XZ, and ZZ contributed to investigation. YW contributed to methodology. CG and JH contributed to conceptualization, resources, supervision, and writing—review and editing. All authors contributed to manuscript revision, read, and approved the submitted version.

## Funding

This work was supported by the National Key R&D Program (2018YFD0900301-07), the Key R&D Program of Hebei Province, China (21326307D), the National Marine Genetic Resource Center, the Natural Science Foundation of Hebei Province (C2019204360) and the Science and Technology Project of Hebei Education Department (QN2020132).

## Conflict of interest

The authors declare that the research was conducted in the absence of any commercial or financial relationships that could be construed as a potential conflict of interest.

## Publisher’s note

All claims expressed in this article are solely those of the authors and do not necessarily represent those of their affiliated organizations, or those of the publisher, the editors and the reviewers. Any product that may be evaluated in this article, or claim that may be made by its manufacturer, is not guaranteed or endorsed by the publisher.

## Supplementary material

The Supplementary material for this article can be found online at: https://www.frontiersin.org/articles/10.3389/fmicb.2023.1178575/full#supplementary-material

## Footnotes

^1^https://github.com/cjfields/readfq

^2^http://bowtiebio.sourceforge.net/bowtie2/index.shtml

^3^http://soap.genomics.org.cn/soapdenovo.html

^4^http://topaz.gatech.edu/GeneMark/

^5^http://www.bioinformatics.org/cd-hit

^6^https://github.com/bbuchfink/diamond/

^7^https://www.ncbi.nlm.nih.gov/

^8^http://www.kegg.jp/kegg/

^9^http://eggnogdb.embl.de/#/app/home

^10^http://www.cazy.org/

^11^https://card.mcmaster.ca/
