# Supplementary material for: Population response of intestinal microbiota to acute Vibrio alginolyticus infection in half-smooth tongue sole (Cynoglossus semilaevis)
Source: Front Microbiol. 2023 May 15;14:1178575. doi: 10.3389/fmicb.2023.1178575 (PMC10275075; doi:10.3389/fmicb.2023.1178575)
Supplement: Supplementary file 2 [file Data_Sheet_2.zip › Figure Captions.pdf]

Figure S1. Images of Half-smooth tongue sole infected with *Vibrio alginolyticus*.

Figure S2. The abundance of non-redundant genes in different-dose groups at different times.

Figure S3. The quantification of 16S rRNA and *groEL* gene by qPCR in transcriptional level. (A) the relative levels of 16S rRNA from *Vibrio* genus in intestinal samples. Significant differences ($P < 0.05$) of 16S rRNA level between the different groups at a given time are indicated with different letter. (B) the expression levels for *groEL* gene of *Vibrio alginolyticus* in intestine. At 24 h and 72 h, *groEL* gene of *V. alginolyticus* was only found in H group. For the calculation with the $2^{-\Delta\Delta Ct}$ method, Ct of the C group was assigned a value of 35.

Figure S4. The biomarkers of gut microbiota analyzed by LEfSe. (A) Circular cladogram presents the differentially abundant taxa in class, order, and family levels. The biomarkers with LDA scores above 3.0 are represented by different colored dots in the cladogram. (B) The histogram presents the differentially abundant taxa in the family, genus, and species levels.

Figure S5. Heat map shows the numbers of genes based on KEGG pathway annotation. The corresponding functional groups are shown on the right. Samples beginning with capital C are from the control group, L from the low dose group, M from the moderate dose group, and H from the high dose group.

Figure S6. Two−component system based on KEGG pathway annotation. The red box represents enzymatic reactions common to both groups, the blue box represents enzymatic reactions unique to the C group at 24 h, and the green box represents enzymatic reactions unique to the H group at 24 h.

Figure S7. ABC transporter based on KEGG pathway annotation. The red box represents enzymatic reactions common to both groups, the blue box represents enzymatic reactions unique to the C group at 24 h, and the green box represents enzymatic reactions unique to the H group at 24 h.

Figure S8. Quorum sensing based on KEGG pathway annotation. The red box represents enzymatic reactions common to both groups, the blue box represents enzymatic reactions unique to the C group at 24 h, and the green box represents enzymatic reactions unique to the H group at 24 h.

Figure S9. Biofilm formation-*Vibrio cholerae* based on KEGG pathway annotation. The red box represents enzymatic reactions common to both groups, the blue box represents enzymatic reactions unique to the C group at 24 h, and the green box represents enzymatic reactions unique to the H group at 24 h.

Figure S10. Bacterial chemotaxis based on KEGG pathway annotation. The red box represents enzymatic reactions common to both groups, the blue box represents enzymatic reactions unique to the C group at 24 h, and the green box represents enzymatic reactions unique to the H group at 24 h.

Figure S11. Flagellar assembly based on KEGG pathway annotation. The red box represents enzymatic reactions common to both groups, the blue box represents enzymatic reactions unique to the H group at 24 h, and the green box represents enzymatic reactions unique to the H group at 72 h.

Figure S12. Heatmap shows the result of CAZy annotation. Samples beginning with capital C are from the control group, L from the low dose group, M from the moderate dose group, and H from the high dose group. The same group of samples are shown in

the same color above the heat map. The taxonomic information of the carbohydrate-active enzymes is shown on the right.
