# Supplementary figures and images for: Population response of intestinal microbiota to acute Vibrio alginolyticus infection in half-smooth tongue sole (Cynoglossus semilaevis)
Source: Front Microbiol. 2023 May 15;14:1178575. doi: 10.3389/fmicb.2023.1178575 (PMC10275075; doi:10.3389/fmicb.2023.1178575)

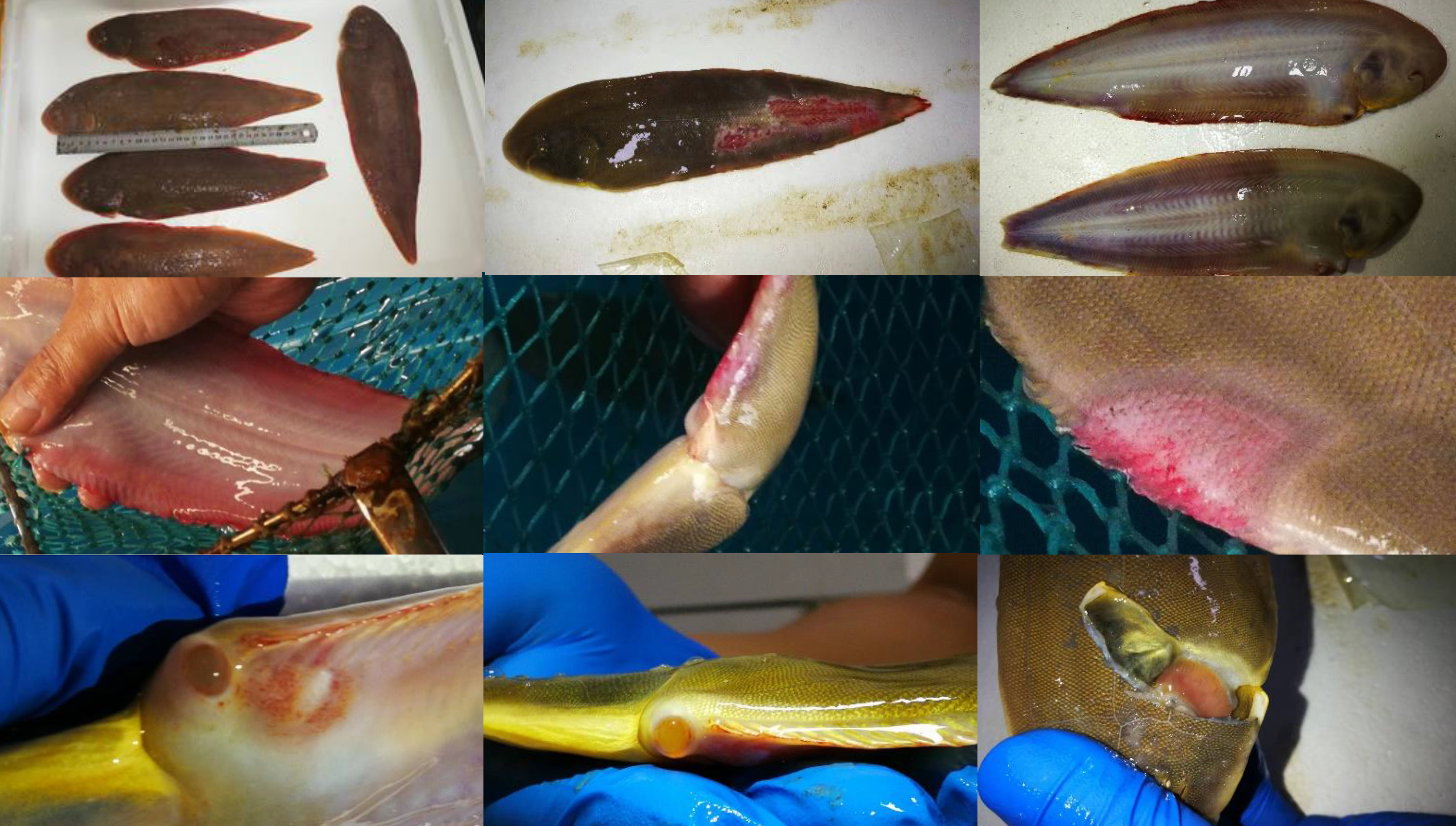

Supplement: Supplementary file 2 [file Data_Sheet_2.zip › Image 1.TIF]

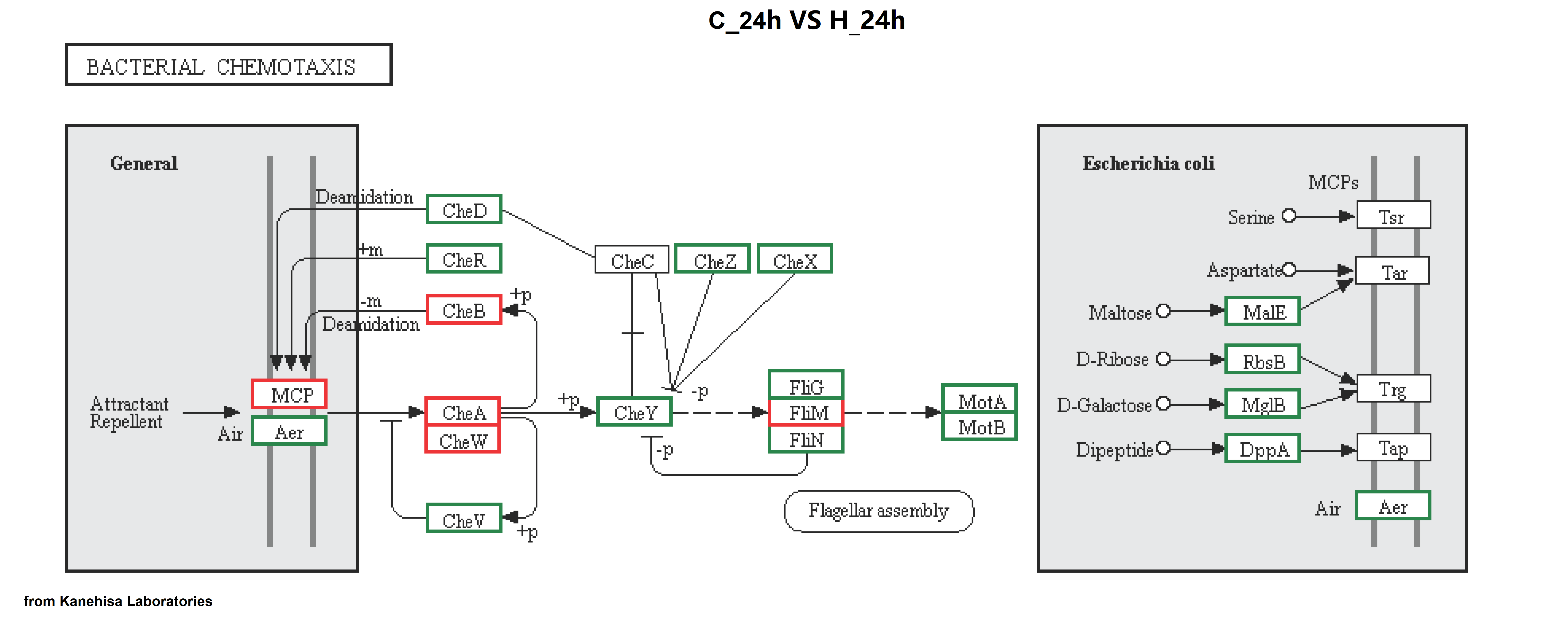

Supplement: Supplementary file 2 [file Data_Sheet_2.zip › Image 10.TIF]

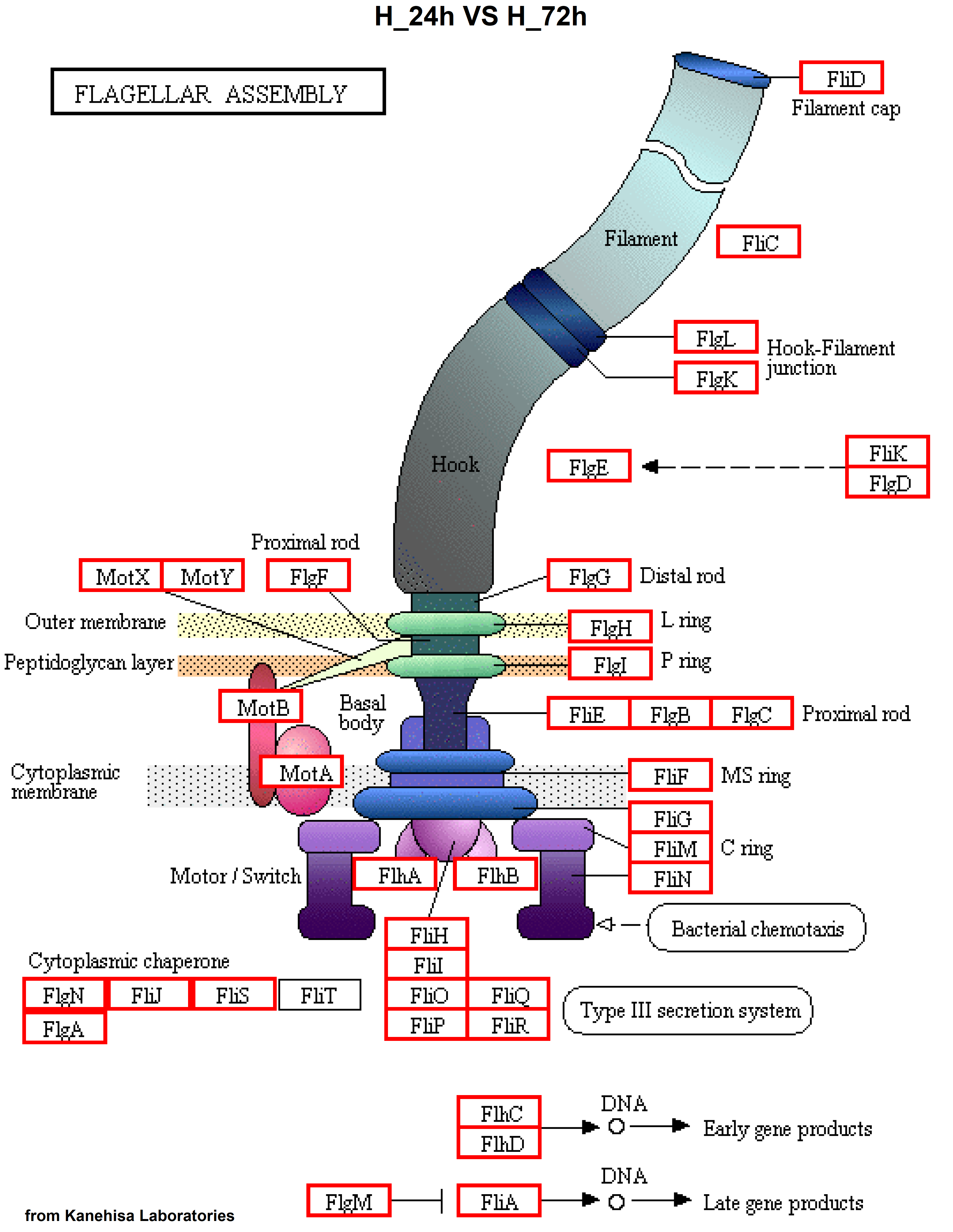

Supplement: Supplementary file 2 [file Data_Sheet_2.zip › Image 11.TIF]

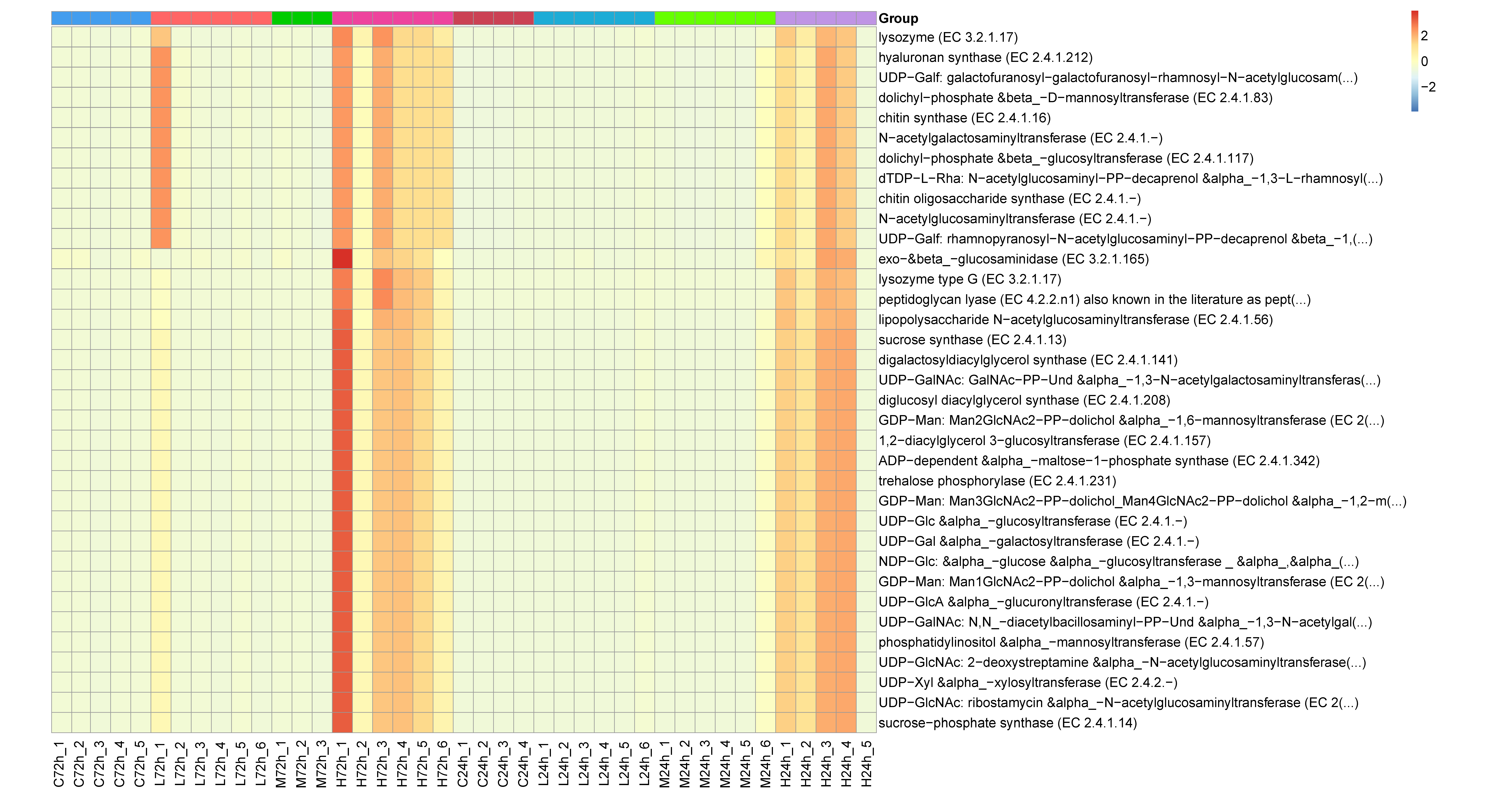

Supplement: Supplementary file 2 [file Data_Sheet_2.zip › Image 12.TIFF]

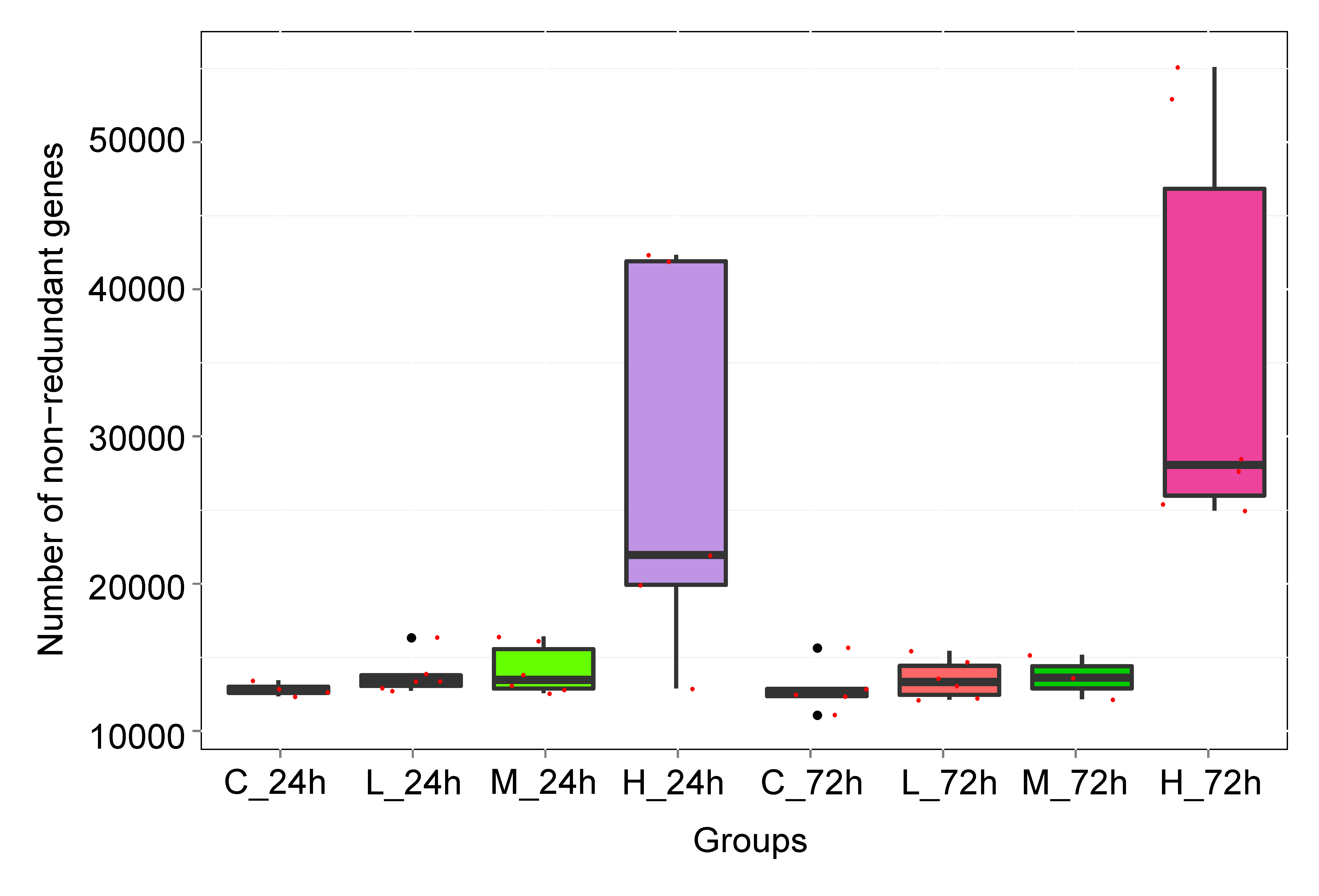

Supplement: Supplementary file 2 [file Data_Sheet_2.zip › Image 2.TIFF]

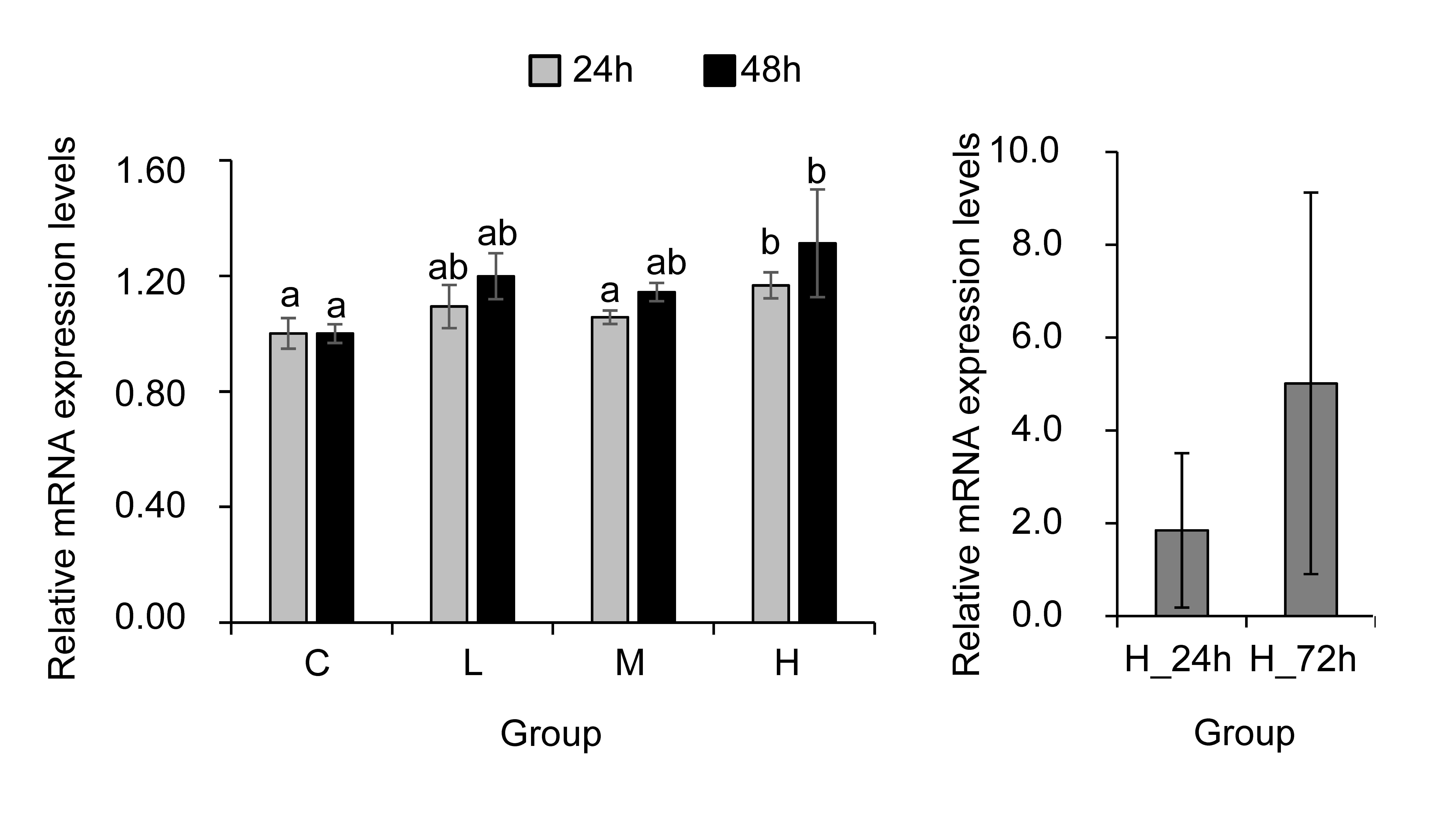

Supplement: Supplementary file 2 [file Data_Sheet_2.zip › Image 3.TIFF]

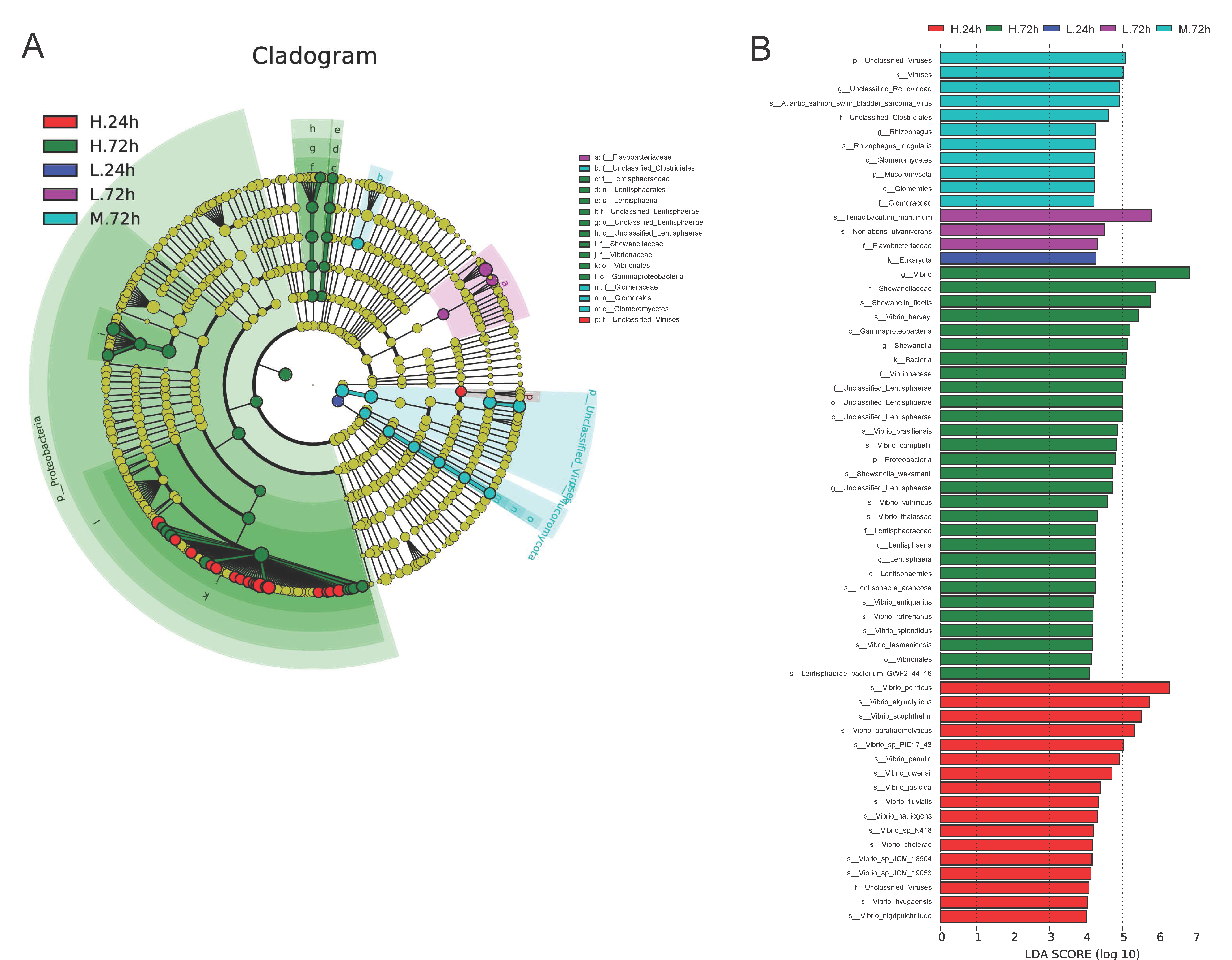

Supplement: Supplementary file 2 [file Data_Sheet_2.zip › Image 4.TIF]

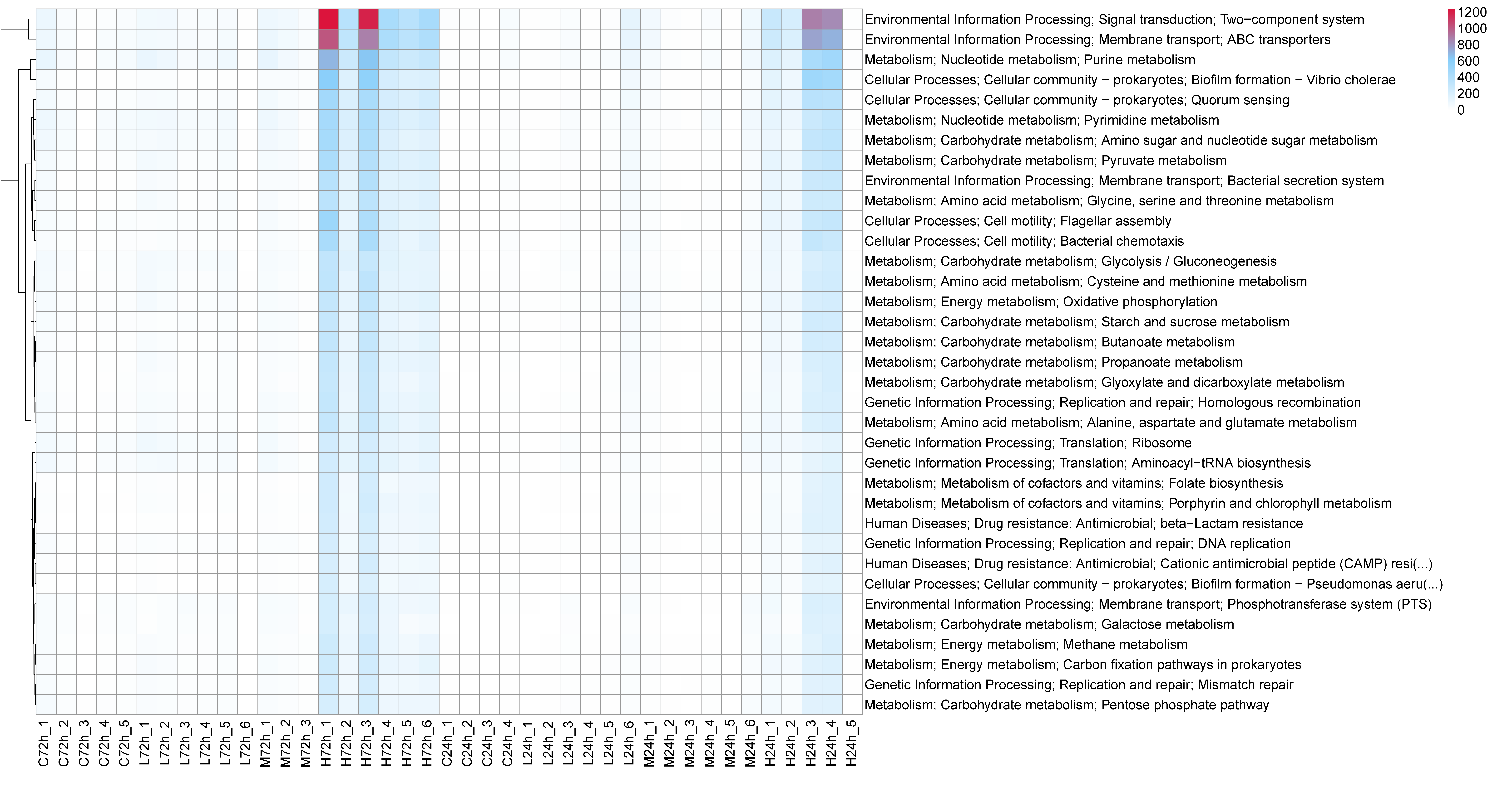

Supplement: Supplementary file 2 [file Data_Sheet_2.zip › Image 5.TIFF]

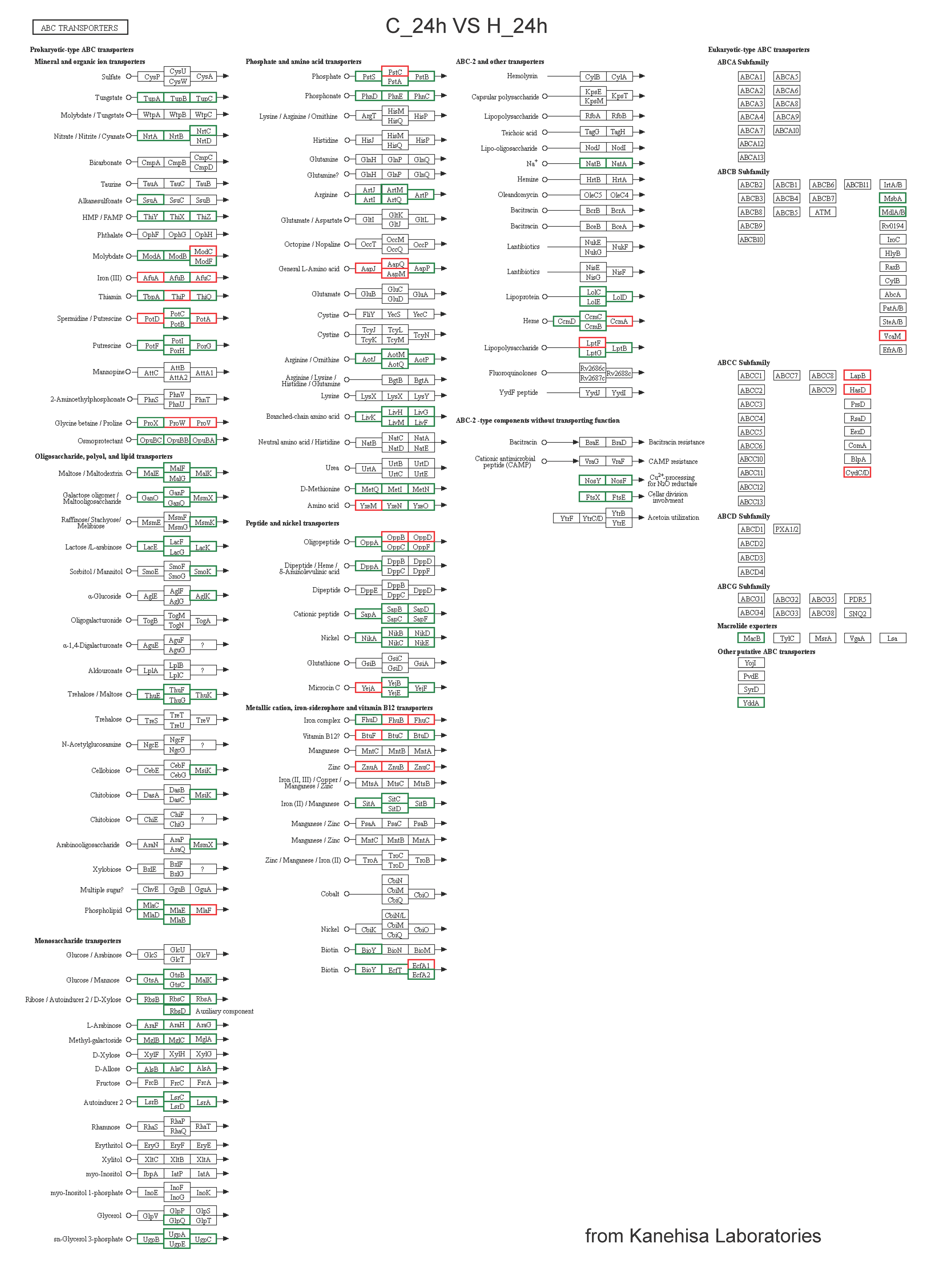

Supplement: Supplementary file 2 [file Data_Sheet_2.zip › Image 7.TIFF]

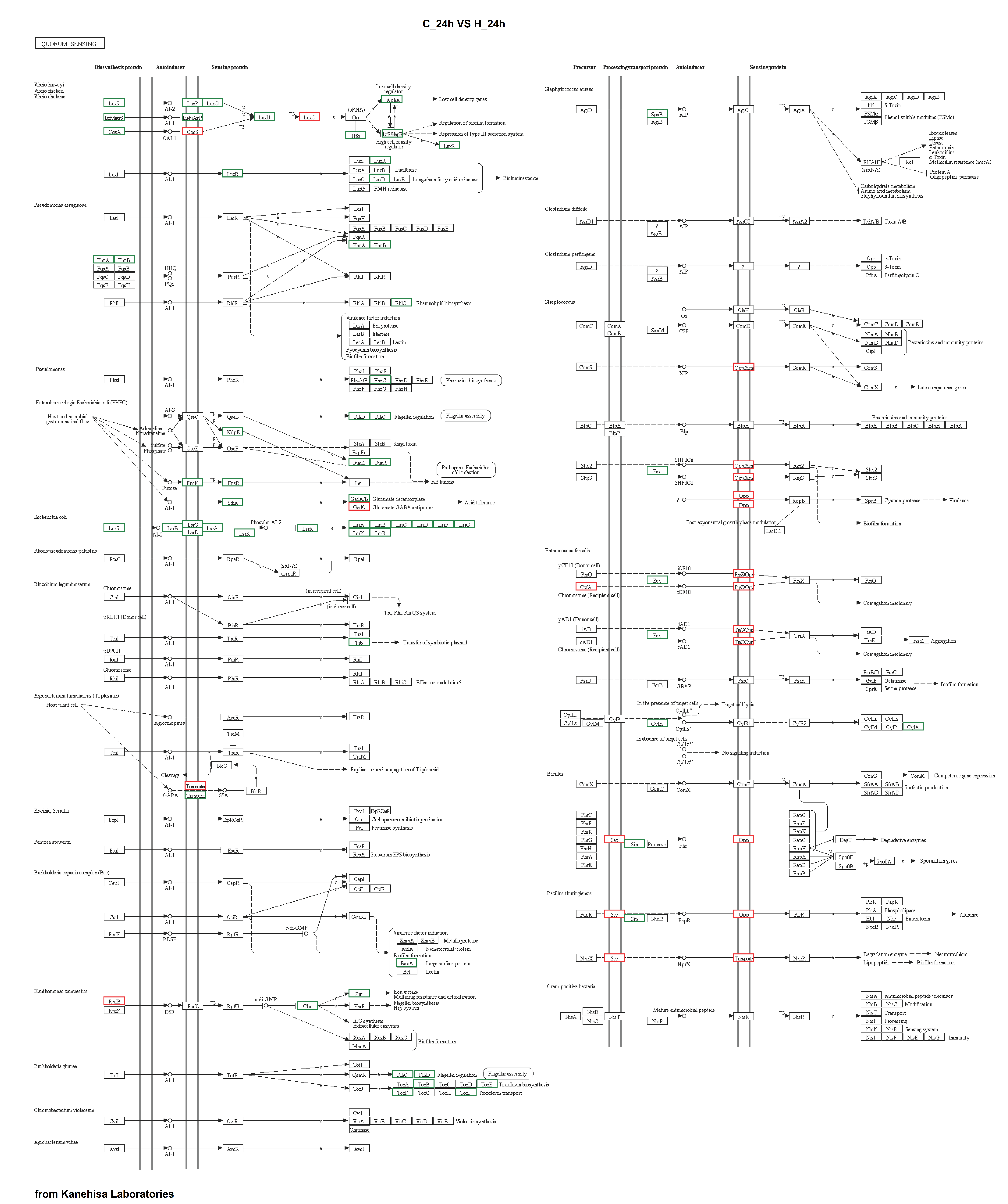

Supplement: Supplementary file 2 [file Data_Sheet_2.zip › Image 8.TIF]

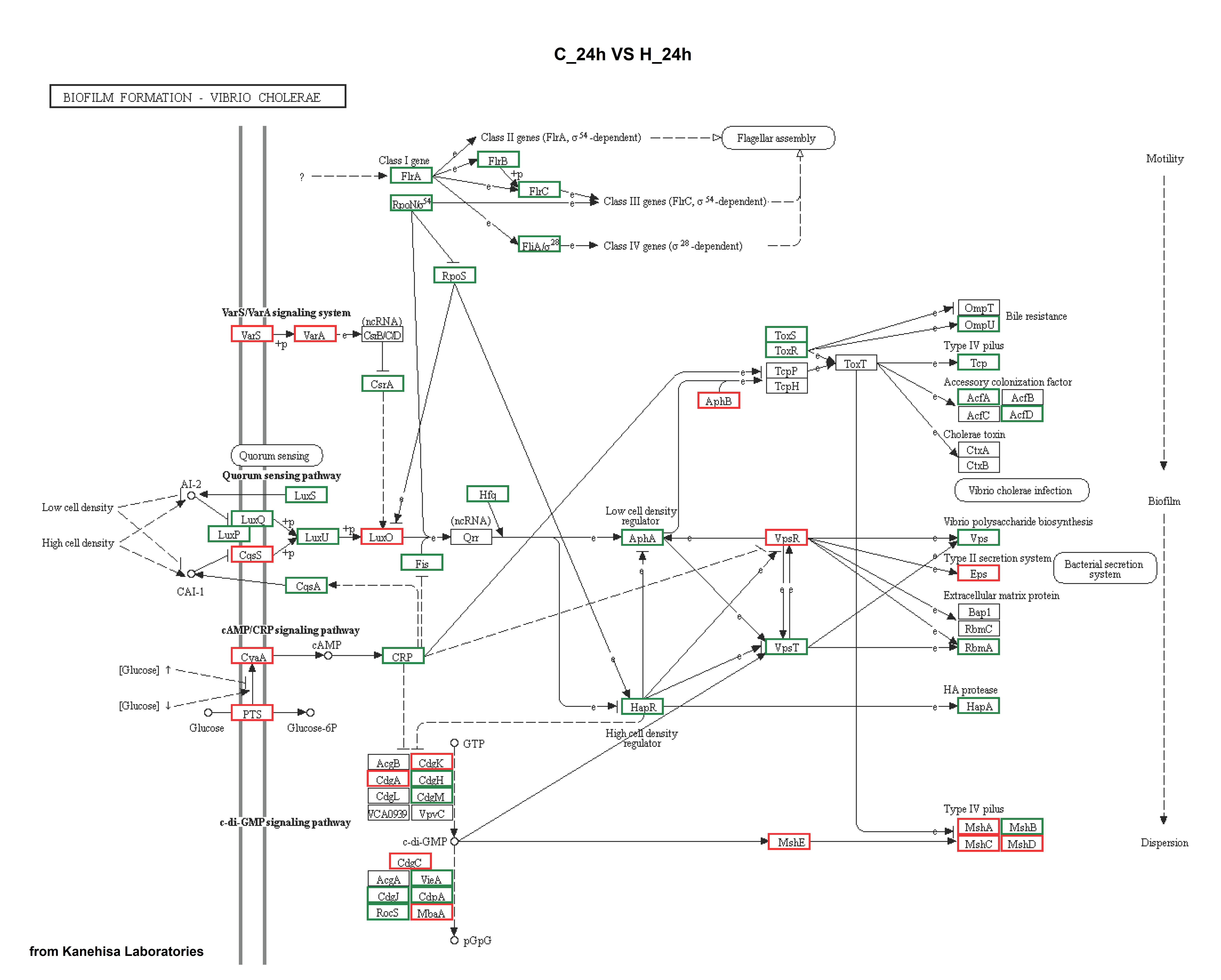

Supplement: Supplementary file 2 [file Data_Sheet_2.zip › Image 9.TIF]
